# From substituent effects to applications: enhancing the optical response of a four-component assembly for reporting ee values†

**DOI:** 10.1039/c5sc04629g

**Published:** 2016-03-07

**Authors:** Chung-Yon Lin, Michael W. Giuliano, Bryan D. Ellis, Scott J. Miller, Eric V. Anslyn

**Affiliations:** a Department of Chemistry, The University of Texas at Austin Austin TX 78712 USA anslyn@austin.utexas.edu +1-512-471-0068; b Department of Chemistry and Biochemistry, College of Charleston 66 George St. Charleston SC 29424 USA; c Department of Chemistry, Yale University 225 Prospect Street, Post Office Box 208107 New Haven CT 06520-8107 USA

## Abstract

High-throughput screening for asymmetric catalysts has stimulated an interest in optically-based enantiomeric-excess (ee) sensors, primarily for their improved time and cost efficiency when compared to the standard HPLC analysis. We present herein substituent-effect studies on a recently reported Zn(ii) multicomponent assembly that is used for chiral, secondary alcohol ee determination. The systematic altering of assemblies formed from select substituted pyridyl ligands pointed to the conclusion that steric effects dominate the mode of interaction at the pyridyl 3- and 6-positions. From these results we identified a new Zn(ii)-centered multicomponent assembly with a higher dynamic range than previously reported. Calibration curves of the CD signals resulting from the new assembly led to an ee assay with a 1.7% error. To further the utility of the new assembly, a correlation was developed between alcohol substituent size to the respective enantiopure CD value.

## Abstract 2

Substituent effect studies were conducted on a dynamic chiral alcohol sensor, leading to a new sensor with lower errors.

## Graphical abstract

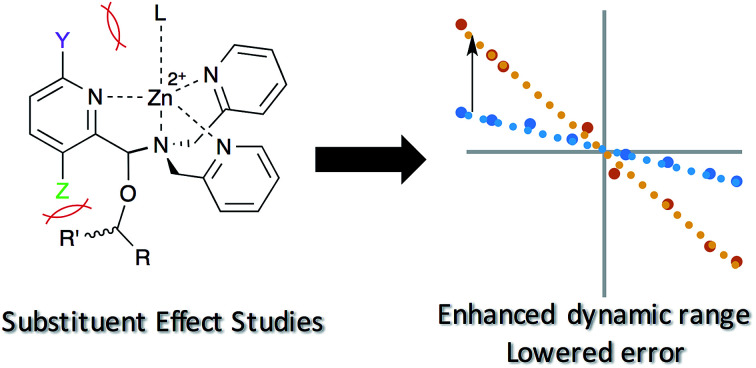

## Introduction

The rapid determination of absolute configuration and enantiomeric excess (ee) for chiral molecules has been a bottleneck for high-throughput screening (HTS) of chiral catalysts.^1^ Currently, the most commonly used methods for enantiomeric excess determination are high performance liquid chromatography (HPLC) and supercritical fluid chromatography, both using chiral stationary phases.^2–4^ Although chiral chromatographic methods are often highly accurate, with error averaging around ±1% for rigorously optimized cases, the major drawback of these methods is their speed and cost.^5^ Techniques such as serial injection and multiplexing have significantly improved the analysis time,^6–8^ but they require additional instrumentation. Subsequently, a wide variety of methods that utilize alternative protocols amenable to HTS are being developed.^9–18^

**Fig. 1:**
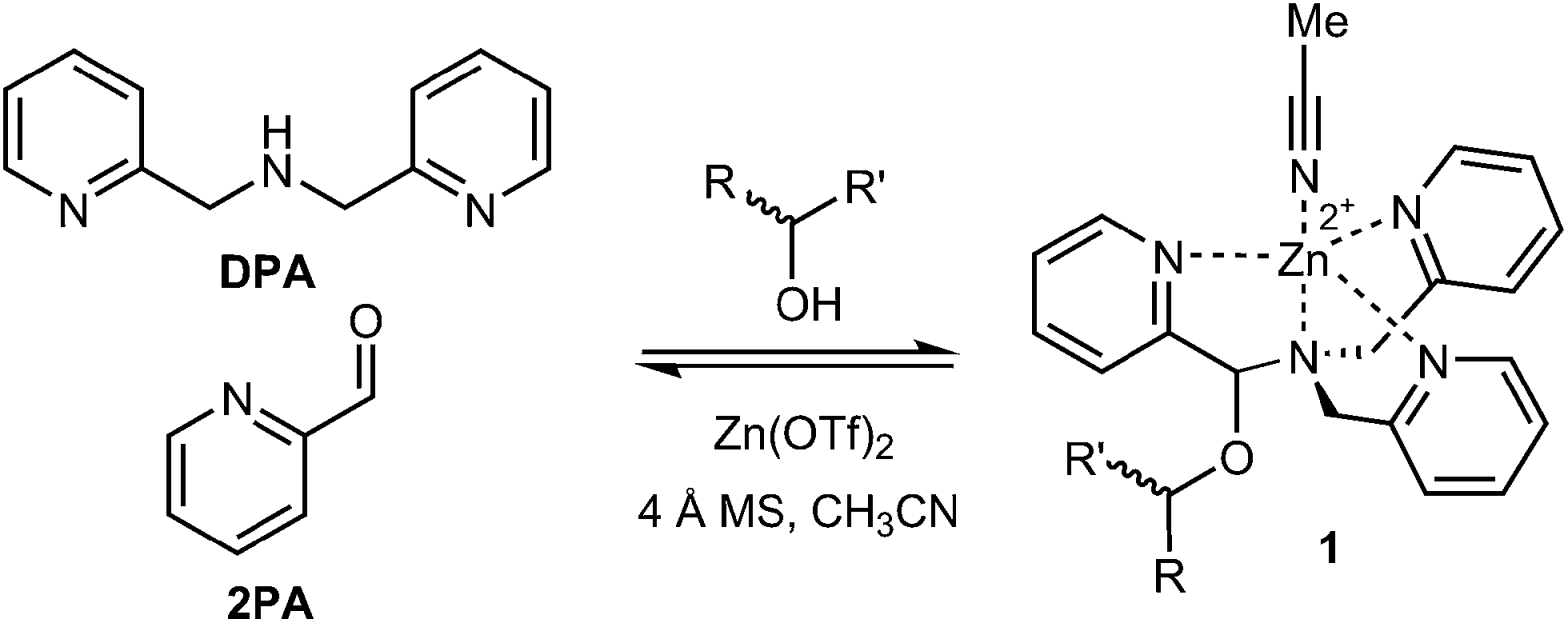
Multicomponent hemiaminal ether assembly (1), formation from 2-pyridinecarbaldehyde (2PA), di(picolyl)amine (DPA), Zn^2+^, molecular sieves (3 Å), and a chiral alcohol analyte.

Sensors based on optical spectroscopic techniques are attractive due to their short analysis time and low cost. For example, various stereodynamic systems that utilize circular dichroism (CD) for ee determination have been published.^12,13,17^ Recently, our group developed a chiral alcohol sensor involving a multicomponent assembly that incorporates the alcohol into a hemiaminal ether (1) under equilibrium conditions^13–15^ (Fig. 1). The incorporation of a chiral alcohol influences the trispyridyl ligand helicity of 1. Because the enantiomers of the alcohol induce opposite twists, their inverse exciton coupled CD (ECCD) spectra enable absolute configuration designation. Furthermore, a calibration curve of this multicomponent assembly determined ee with average ±3% absolute error.

**Fig. 2:**
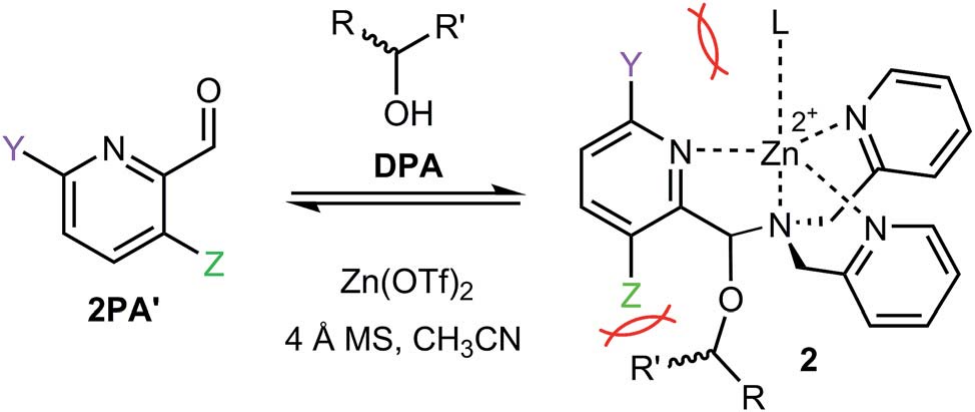
3- or 6-substituted 2PA (shown as 2PA′) were hypothesized to alter the multicomponent assembly (2) differently leading to altered dr and CD. The 3-substituent (represented by Z) introduces steric bulk proximal to the alcohol substrate while the 6-substituent (Y) interacts with the axial metal ligand L.

With the success of multicomponent assembly 1, we turned our attention to improve the analytical power of the technique. One major limitation of this original assembly was a low CD intensity for chiral alcohols with similar sized substituents at the stereocenter. When an alcohol substrate has similar substituents, a slight preference toward one tris-pyridyl helicity is observed, which results in a small dynamic range for the CD ellipticities and an increased error in ee determination. To counter this problem, we investigated the effect of 3- and 6-substituents on the heterocyclic ligand 2PA. Given the tripodal geometry of the assembled complex 1, it was hypothesized that, due to proximity to the hemiaminal ether, altering the 3-position substituent (Fig. 2, Z) would enhance the assembly sensitivity to the differences in the steric size of the alcohol substrates, thereby increasing the diastereomeric ratio (dr) values. Additionally, the 6-substitution (Fig. 2, Y) was expected to similarly alter the dr values due to different interactions with the axial metal ligand (L). Because we have previously found that larger dr values result in larger CD signals and lower errors in ee determination,^13–15^ the goal of our substitutent effect studies was to enhance the dr's.

## Results and discussion

The effects of 3- and 6-substituents were examined in assemblies formed with different 2PA derivatives (2PA′). The conditions for assembly formation followed closely that of the previously published protocol.^13^ Each 2PA′ (1 equiv. at 35 mM) was mixed with DPA (1.2 equiv.), followed by addition of Zn(OTf)_2_ (1 equiv.), molecular sieves (3 Å), and 4-(2-chloroethyl)morpholine HCl (1 equiv.) in acetonitrile at 35 mM. (*R*)-1-Phenylethanol (3 equiv.) was used as the standard analyte. Rather than 18 hours at room temperature equilibration as previously reported, it was found that incubating the assembly for 1 hour at 40 °C yielded the same result. Thus, all the multicomponent assembly experiments described herein presumed to reach equilibrium in this manner.

Each new assembly was characterized by ^1^H NMR and CD (ESI†). Additionally, dr's and yields of the assemblies were calculated by ^1^H NMR. Assembly 2 exists as diastereomers, and the dr value is defined as the ratio of the major and minor diastereomer at equilibrium, while the yield is defined as the extent of formation of 2 (see ESI† for an example). Note, the yield here is not isolated yield but rather a measure of thermodynamic preference for the hemiaminal ether assemblies. Systematic examination of CD, yield, and dr values allowed us to gain insights on the properties of these multicomponent assemblies. While CD is the ultimate signal response of interest for ee determination, the assembly yield represents a measure of the relative thermodynamic preference for formation of the hemiaminal ether complexes, while the dr value provides information on the relative stabilities of the two diastereomers formed in each assembly.

The assembly yield and dr values are shown in Table 1. Low extents of assembly formation (<5%) were observed for quinoline-2-carbaldehyde (QA) and phenanthridine-6-carbaldehyde (PNA). The lack of complex formation was attributed to the aromatic hydrogen blocking the coordination of the Zn metal (Fig. S1†). Although this is a thermodynamic issue inhibiting assembly, it is in accord with our recent finding of the involvement of Zn(ii) in the rate determining step of assembly formation.^19^ In the instance of no such proton obstruction, the assembly formed with isoquinoline-1-carbaldehyde (IQA) and exhibited similar CD, dr, and yield as 2PA. However, due to the comparable or lower performances of these ligands, we turned our attention to non-benzofused substituents.

**Table 1:** Multicomponent assembly hemiaminal ether yield and dr formed with various pyridine carbaldehyde derivatives (2PA^3^, 2PA^6^, IQA, QA, 2PA, and PNA) and 1-phenylethanol^a^

Code	Substituent	2PA^3^ assembly		2PA^6^ assembly	
		Yield	dr	Yield	dr
2PA′^F^	F	87%	1.51	73%	1.45
2PA′^Cl^	Cl	87%	1.62	58%	1.71
2PA′^Br^	Br	86%	1.92	52%	1.61
2PA′^MeO^	MeO	96%	1.41	52%	1.89
2PA′^Me^	Me	97%	2.01	83%	2.28
2PA	H	83%	1.41	83%	1.41
IQA	3,4-Benzo	79%	1.45	n/a	n/a
QA	5,6-Benzo	<5%	n/a	n/a	n/a
PNA	Dibenzo	<5%	n/a	n/a	n/a

a The 3 and 6 subscribes designate the regiochemistry of PA substitution.

All of the non-benzofused ligands (Table 1) formed the multicomponent assembly. However, the assemblies formed with 3-substituted ligands (2PA^3^) consistently outperform in yield their 6-substituted counterparts (2PA^6^). This discrepancy potentially carries the same explanation for why QA and PNA give poor yields; an alteration at the 6 position introduces steric bulk that hinders Zn(ii) coordination.

**Fig. 3:**
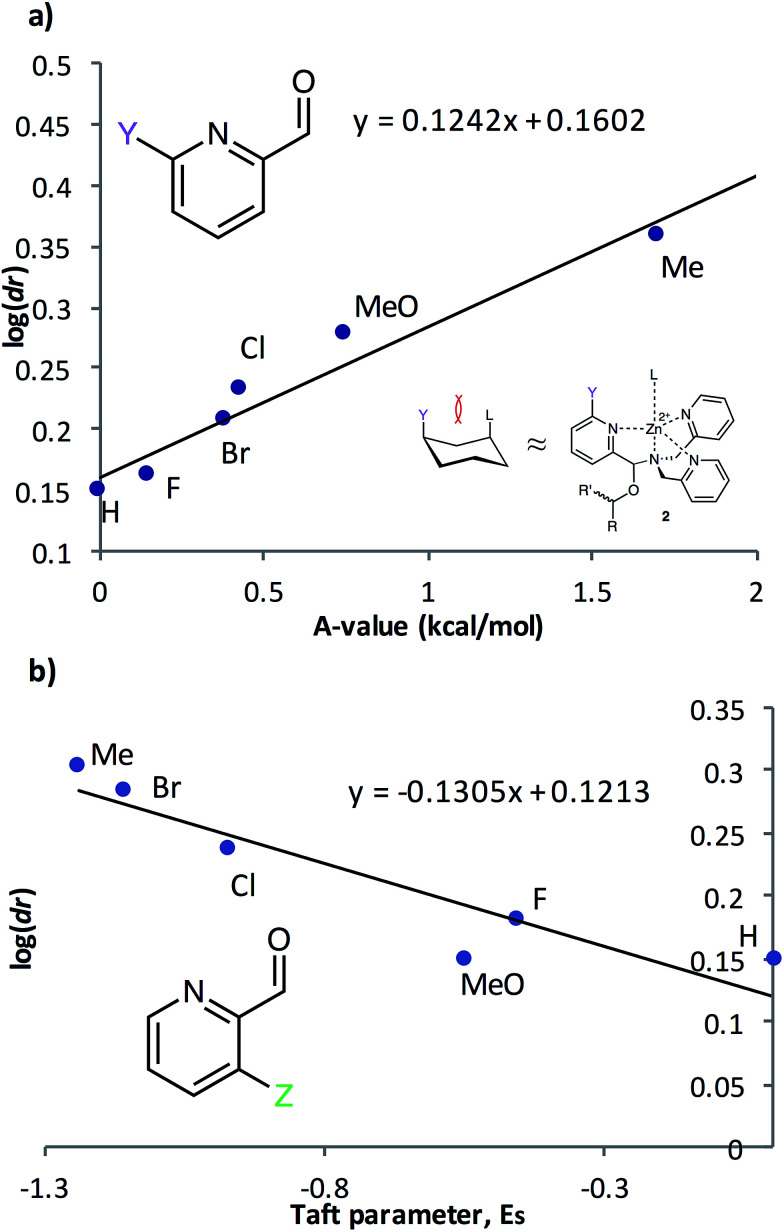
Linear plots showing log(dr) values for assemblies involving (a) 6-substituted pyridine-2-carbaldehydes plotted against *A*-values corresponding to the substituents, and (b) 3-substituted pyridine-2-carbaldehydes plotted against corresponding Taft steric parameters.

To further understand the nature of these substituent effects, the observed assembly dr was correlated with linear free energy relationships (LFERs) (Fig. 3). Because the interaction between ligands 2PA^6^ and L resembles the 1,3-diaxial interaction of substituted cyclohexanes,^20^*A*-values were used as the corresponding substituent parameter (Fig. 3a). A linear correlation (*R*^2^ = 0.95, slope = 0.12) was observed between log(dr) and *A*-values. The strong linearity affirms that the assembly responds to changes in substituent in the same manner as the cyclohexane system, but that the assembly is 12% as sensitive as cyclohexane to substituent changes. Similarly, 2PA^3^ correlated linearly (*R*^2^ = 0.86, slope = −0.13) with Taft steric parameters (Fig. 3b) with approximately 13% the sensitivity as the substituent changes with respect to the reference reaction. These linear correlations with two different steric LFERs affirmed our hypothesis that there are two different modes of steric effects in the multicomponent assembly.

**Fig. 4:**
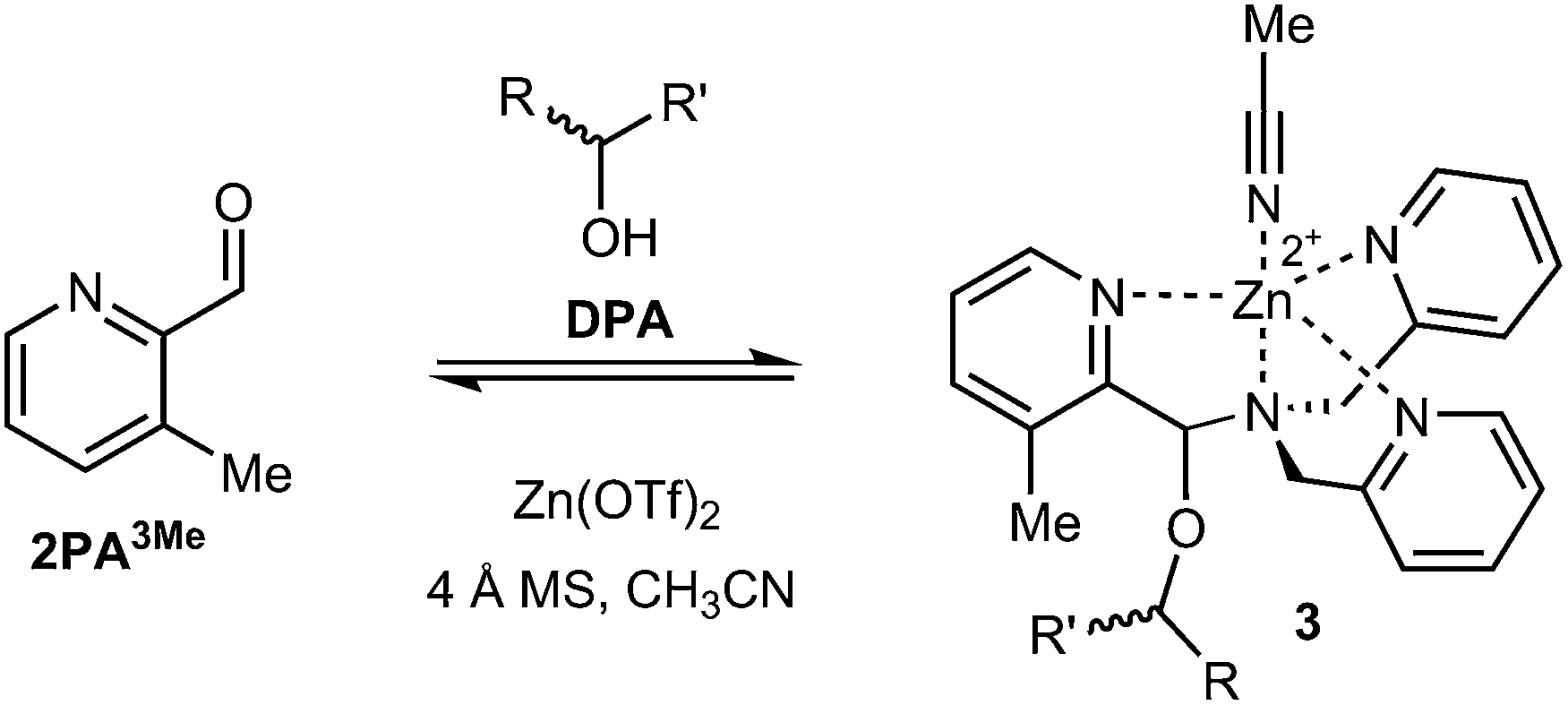
Multicomponent assembly 3 formed with ligand 2PA^3Me^, DPA, Zn(OTf)_2_, and chiral alcohol following the same protocol as reported above.

Following the substituent effect studies, 3-methylpyridine-2-carbaldehyde (2PA^3Me^) was the ligand that most significantly improved the assembly dr (Fig. 4), with bromide as a close second (2PA^3Br^). Thus, we expected the CD signals for assemblies using 3-Me and 2-Br to be similar. However, broadening of the ECCD signal was observed with Br due to the nature of exciton coupling, where the signal originates from the coupling of excited chromophores. When the chromophores participating in ECCD are identical, a sharp couplet is observed. If the three participating pyridyl chromophores do not share identical absorbance spectrum, a broadening in the ECCD signal is observed^21^ (ESI†), as is evident for Br substitution. However, methyl does not alter the absorbance of pyridine significantly, and therefore the ECCD remains sharp. Efforts to form the assembly with matching di- and tri-substituted bis-3-methyl and bis-3-bromo DPA-like ligands were unsuccessful, likely due to steric limitations (Fig. S2†).

**Fig. 5:**
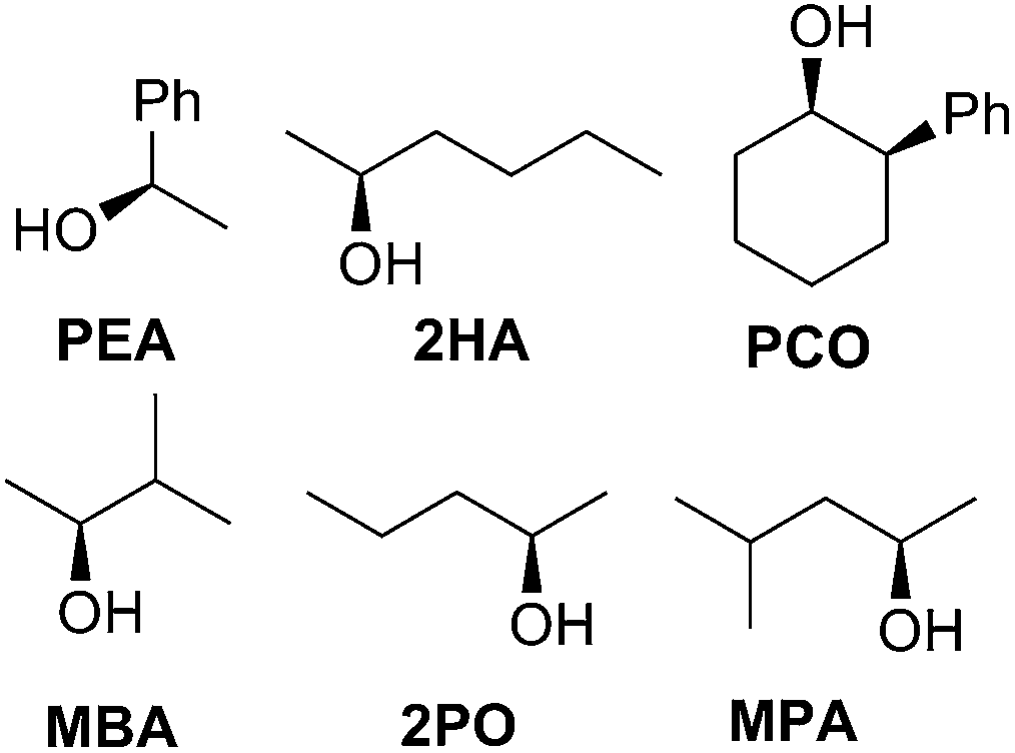
The alcohols used in the correlating alcohol steric size to their corresponding CD signal.

**Fig. 6:**
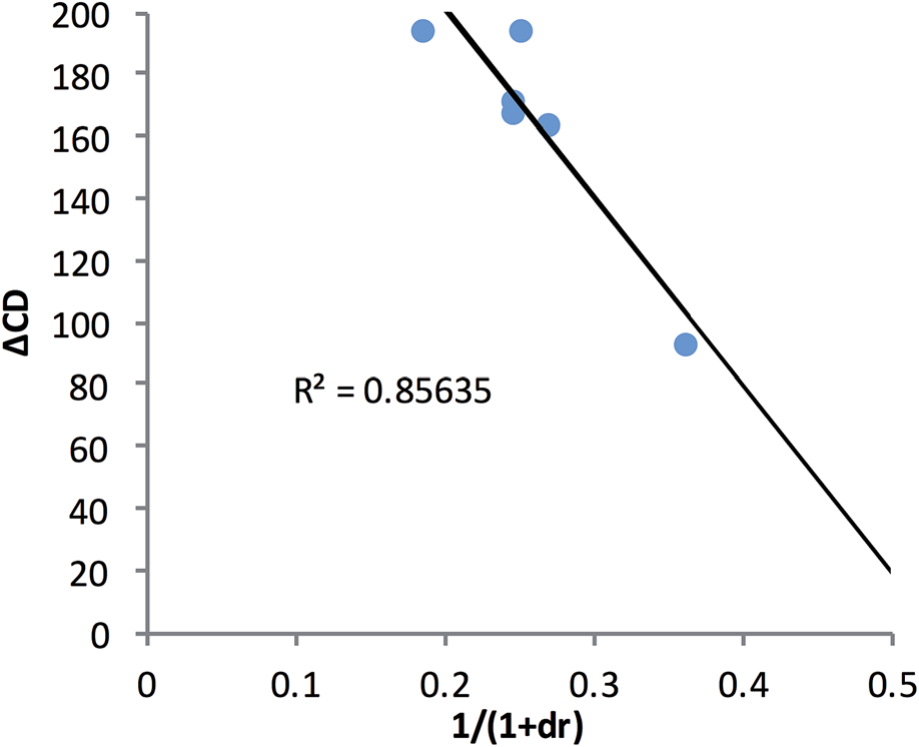
The alcohol dr value was correlated to their enantiopure sample CD value at 270 nm using assembly 3.

In an effort to extend the utility of assembly 3, a model was developed to correlate the ECCD signal and dr values to the steric size of the groups on the stereocenter of chiral alcohols. Six alcohols were chosen to cover aromatic, cyclic aliphatic, linear alkyl, and branched alkyl side chains (Fig. 5). First, it should be noted that the CD signal for the assembly has an inherent maximum, because the pyridine rings in 3 can twist only to a certain extent before the ligand no longer binds Zn(ii) and the complex disassembles. Therefore, the magnitude of the CD does not correlate to dr value linearly, but rather by a half sigmoid (Fig. S3†). That is, the CD intensity approaches a maximum asymptote as the dr value approaches infinity, and conversely, as the dr approaches one, the signal drops to zero. Given this logic, eqn (1) was developed where ΔCD is the difference between CD signals at 270 nm for enantiopure *R* and *S* samples of the chiral alcohols and CD_max_ is the theoretical CD maximum of an assembly.^15^ For all the alcohol samples, dr values were plotted against ΔCD (*R*^2^ = 0.86, Fig. 6). The plot predicts a maximum CD of 186.6 mdeg, for 3, which is significantly higher than the reported maximum CD for the original assembly 1 (113.5 mdeg).1
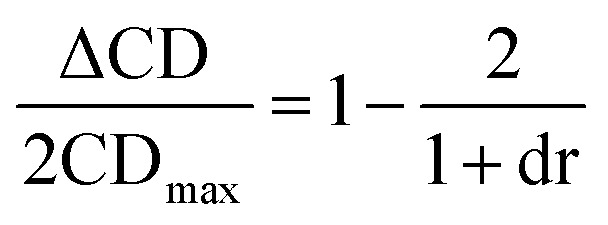


Once a linear relationship between dr and CD had been established, we turned our attention to correlate alcohol sterics to their corresponding assembly dr value. Charton parameters were used to calculate the absolute value of the difference in the size of the non-hydrogen substituents (Δ*v*) on the stereogenic α-carbon of each alcohol (Table 2).^22^

**Table 2:** The difference in substituent Charton parameter (Δ*v*) and their corresponding hemiaminal ether dr value for 3

Alcohol	Δ*v*	dr
PCO	0.57	3.02
MPA	0.46	4.38
2HA	0.16	2.97
2PA	0.16	2.67
PEA	0.05	2.01
MBA	0.24	3.03

**Fig. 7:**
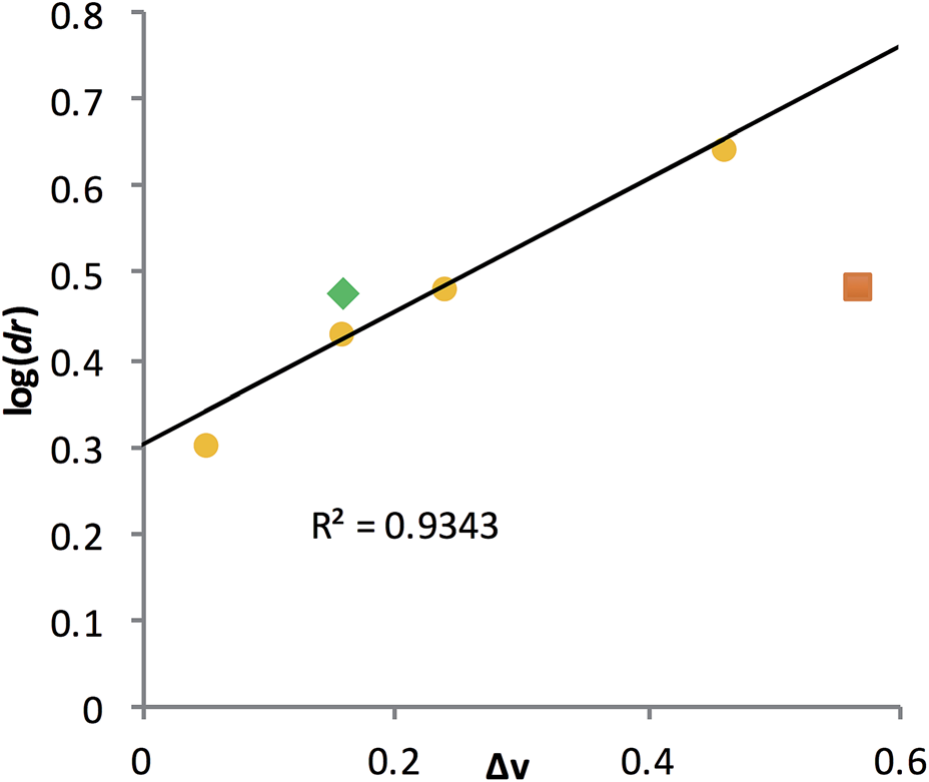
Linear correlation was established between the alcohol substituent Charton parameters (Δ*v*) and their corresponding log(dr) value.

A poor linear correlation (*R*^2^ = 0.49) between Δ*v* and log(dr) of the alcohols was observed, caused by inaccurate estimation in Δ*v* for PCO and 2HA. For the conformationally restricted PCO, considering only the 2 and 6 position substituents overestimates Δ*v versus* the higher degree of freedom alkyl chains. In the case of 2HA, Charton parameters predict the linear butyl substituent to be the same size as the linear propyl substituent (same value for *n*-propyl and *n*-butyl), resulting in 2HA having the same Δ*v* as 2PO. This results in an underestimation of Δ*v* for 2HA. Dramatic improvement in the correlation (*R*^2^ = 0.93, Fig. 7) was observed once PCO (red square in Fig. 7) was removed from the data set, while removing 2HA (green diamond) from the set further improved the correlation (*R*^2^ = 0.99).

**Fig. 8:**
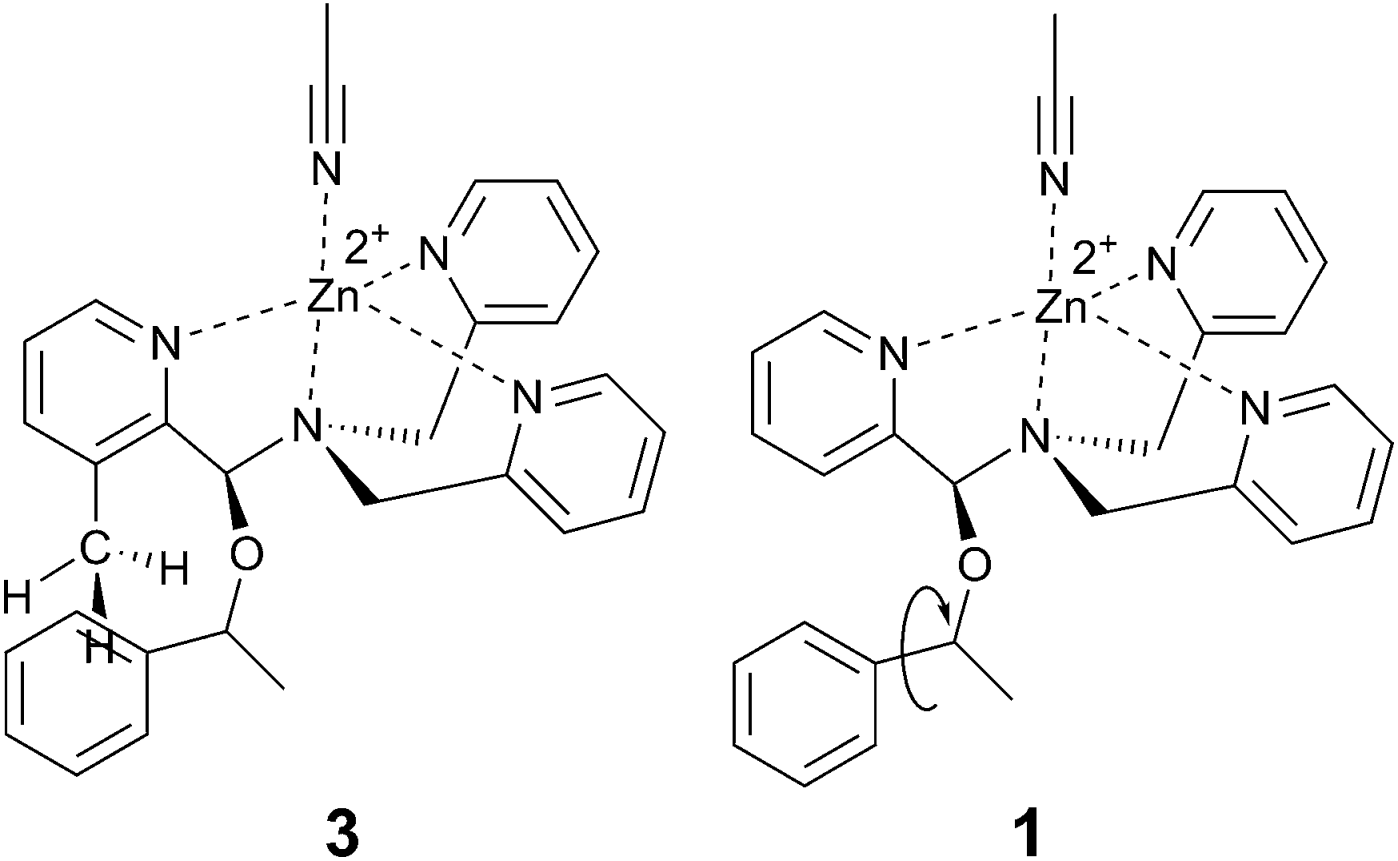
Proposed origin of context dependent steric size. Assembly 3, with an additional proximal methyl group, hinders the alcohol phenyl substituent rotation.

Examining the LFERs involving correlations of phenyl steric size *versus* other substituents on the stereocenter of the alcohol pointed to a curious difference between assemblies 1 and 3. There are two reported steric values for phenyl. The larger Charton value of *v* = 1.73, pertaining to a freely rotating phenyl, was previously applied to the analysis of assembly 1.^13–15^ However, we found that the pyridinyl methyl group in 3 restricts the rotation of the β-phenyl substituents on the alcohol substrates. Therefore, the smaller phenyl Charton parameter, *v* = 0.57, gave a better linear fit to the data in Fig. 7. This smaller steric value describes interactions with a rotationally restricted phenyl ring, such that only one face of the ring is presented to the reactant. Thus, while the original assembly 1 exhibits free rotation of the phenyl group, the congested environment of assembly 3 forces the phenyl substituent to adopt a conformation with minimal steric interactions (Fig. 8 and S4†).

**Fig. 9:**
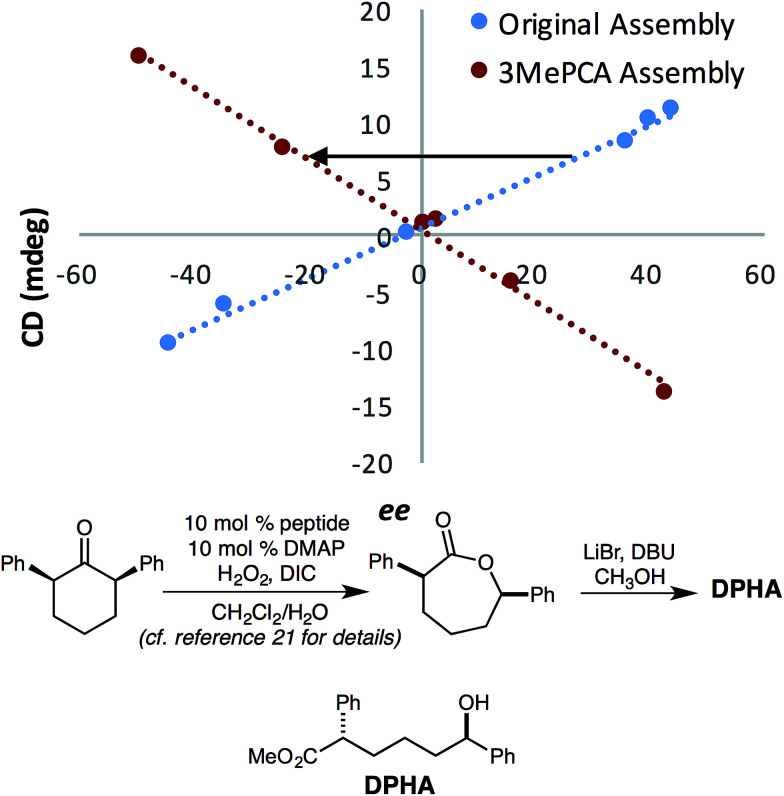
Calibration curves of the previously published alcohol analyte with assemblies 3 and 1, *R*^2^ = 0.99.^21^ The assembly was formed at 35 mM (with 3 equiv. excess of alcohol), and CD at 270 nm was taken with a 175 μM assembly solution in MeCN. The slope of the calibration curve for 3 flipped in comparison to that for 1.

This change in the perceived steric size of a phenyl ring for different assemblies had a further ramification when studying a chiral alcohol we had previously analysed with assembly 1. In recent studies, we had measured the ee values of several catalysts for an asymmetric Baeyer–Villiger reaction, that after lactone hydrolysis, led to a 1-phenyl substituted alcohol (DPHA, Fig. 9).^23^ An opposite Cotton effect in the CD spectra of assembly 3 was observed for the same enantioenriched sample of DPHA as that for assembly 1 (Fig. 9). While initially puzzling, further investigation revealed that assembly 3 recognizes a phenyl group sterically as between a methyl (*v* = 0.52) and an ethyl (*v* = 0.56) group. Assembly 3 reverses the helical twist of the pyridine rings when a methyl is changed to an ethyl in a 1-phenyl alkanol chain (Fig. S4†). Further increase in the chain length continues to increase the magnitude of the CD values, but still with a negative Cotton effect for assembly 3. This effect is evident even though the Charton parameter for phenyl is similar to ethyl (0.57 *vs.* 0.56). Thus, we find another example that steric size is context dependent, and the Charton parameters do not perfectly predict the size differences of the groups on the stereocenters of the chiral secondary alcohols within the context of 3. In fact, a closer examination of phenyl substituted alcohols in the linear model leads to similar conclusion (Fig. 7, shown in blue).

**Fig. 10:**
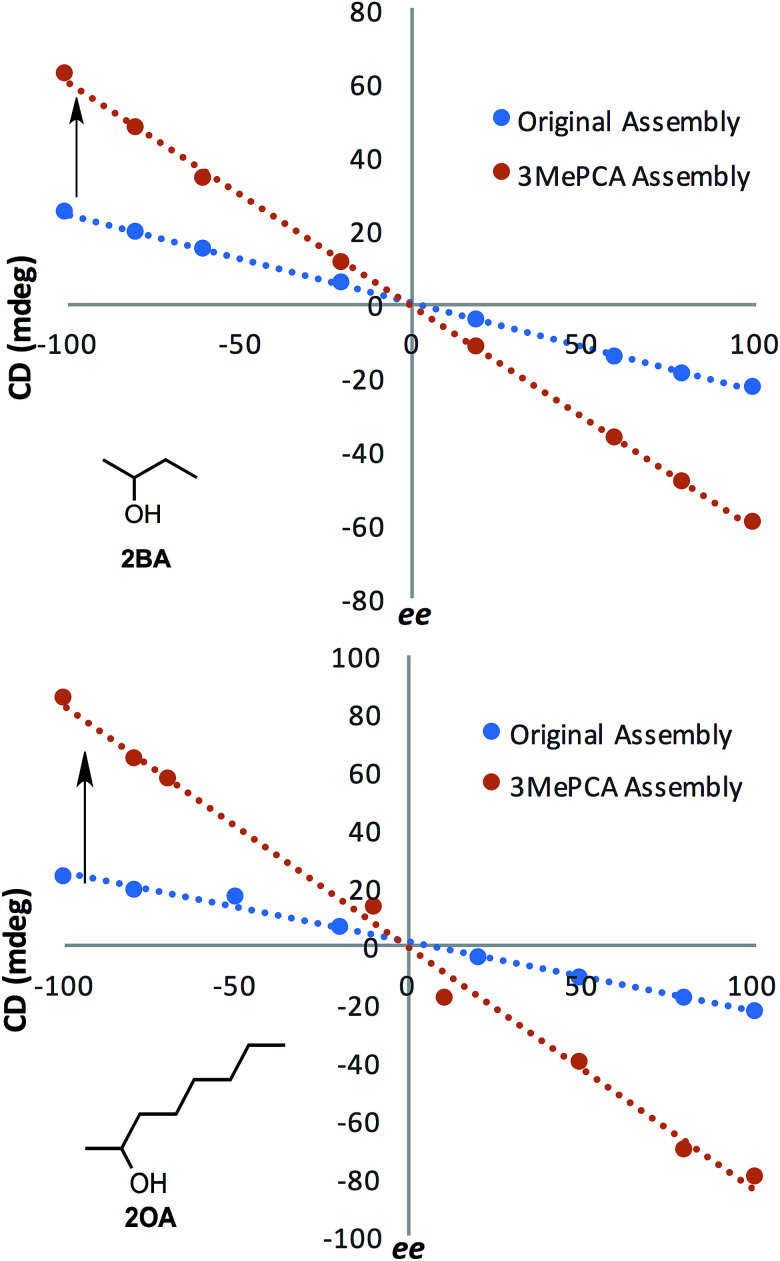
Linear calibration curves of 2OA and 2BA with assemblies 1 and 3 were constructed with maximum cotton effect CD at 270 nm (*R*^2^ = 0.99). The assemblies were formed at 35 mM (with 3 equiv. excess of alcohol), and CD was taken with a 175 μM assembly solution in MeCN. Assembly 3 calibration curves showed an increase in dynamic range in comparison to their original assembly counterparts.

After developing models that correlate analyte steric size to their corresponding ECCD signals in assembly 3, we shifted our focus to demonstrate the enhanced dynamic range for ee determination and the corresponding lowering of the error, as was the initial goal of the project. Calibration curves were constructed for alcohols 2OA and 2BA using assembly 3. The results were compared to the original assembly calibration curve (*R*^2^ = 0.99 for all assemblies, Fig. 10). The values of ee ranged from 100% (100% (*R*)-enantiomer) to −100% (100% (*S*)-enantiomer), and were plotted against the signal observed at 270 nm. The optical response to ee using assembly 3 is about 3–4 times as large to that using assembly 1. The 2OA calibration curve for assembly 3 was used to calculate the ee of three blind samples, and the average absolute error was found to be 1.7%. This improvement in error over 1 results from the enhanced dynamic range of 3.

## Conclusions

The studies described herein demonstrate that linear free energy relationships that reflect steric size can correlate the magnitude of the dr values for the 4-component assemblies represented by Fig. 3. The steric sizes of the substituents on the 3- and 6-positions of 2PA as well as the groups on the stereocenter of the alcohol dictate the dr and CD optical response. The dependence on sterics was such that two different steric sizes for phenyl were necessary, depending upon the assembly, to model the data properly. Through these studies, we found the assembly containing 2PA^3Me^ had the most improvement in the dynamic range of the optical response, resulting in lower errors for ee determination.

## Supplementary Material

SC-007-C5SC04629G-s001
