# Supplementary material for: From substituent effects to applications: enhancing the optical response of a four-component assembly for reporting ee values
Source: Chem Sci. 2016 Mar 7;7(7):4085–90. doi: 10.1039/c5sc04629g (PMC5125730; doi:10.1039/c5sc04629g)
Supplement: SC-007-C5SC04629G-s001 [file SC-007-C5SC04629G-s001.pdf]

Electronic Supplementary Material (ESI) for Chemical Science.
This journal is © The Royal Society of Chemistry 2016

Supplementary Information

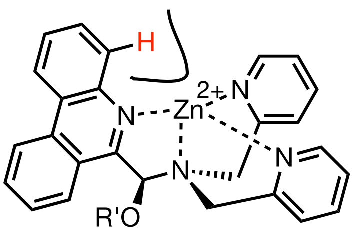

Figure S1. The unfavorable positioning of aromatic hydrogen for 5, 6-benzo fused ligands (PNA shown above) resulted in the lack of assembly formation. The axial metal ligand was omitted for clarity.

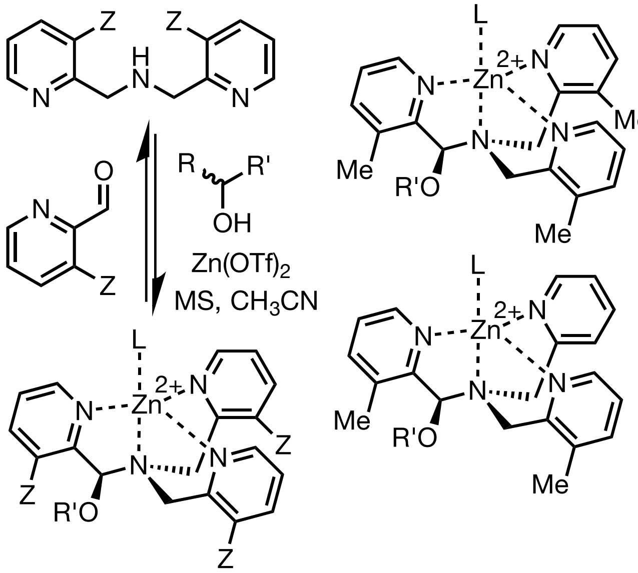

Figure S2. Lack of assembly formation was observed when attempted with di- or tri- substituted ligands. Methyl substituted variants are shown above.

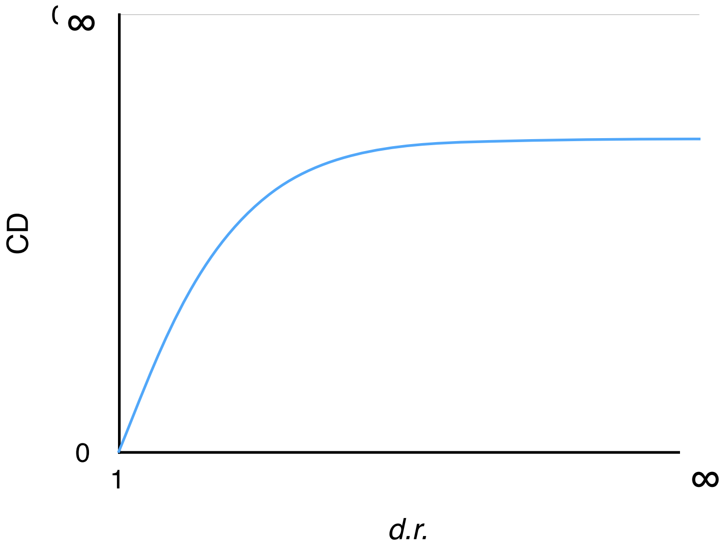

Figure S3. The theoretical relationship between assembly dr values and CD values.

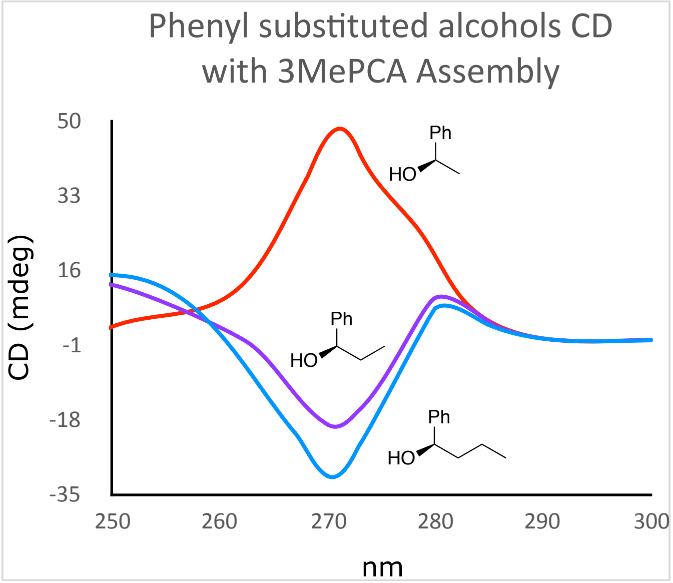

Figure S4. CD spectra of 3MePA with various enantiopure (S)-1-phenyl alcohols. As the alkyl chain grows, the assembly Cotton effect grows in opposite direction indicating the phenyl group size is between a methyl and ethyl substituent.

Assembly Characterization Data

Substituent Effect Studies

^{1}H NMR and CD spectra were used to characterize each multicomponent assembly formation in acetonitrile at 50mM, and the assemblies formation protocol followed the procedure described in the main text.

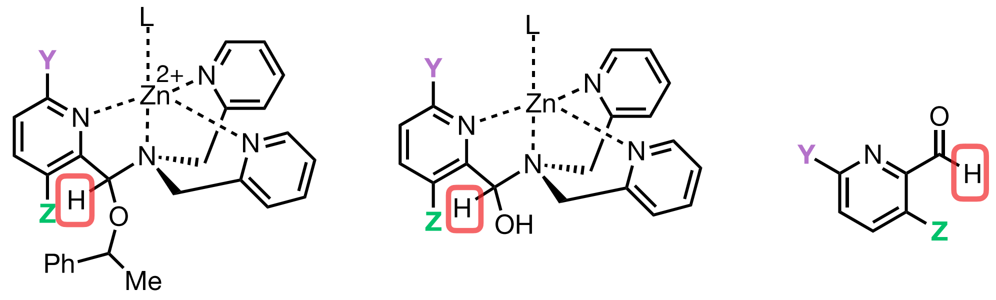

1 **2** **3**

3-

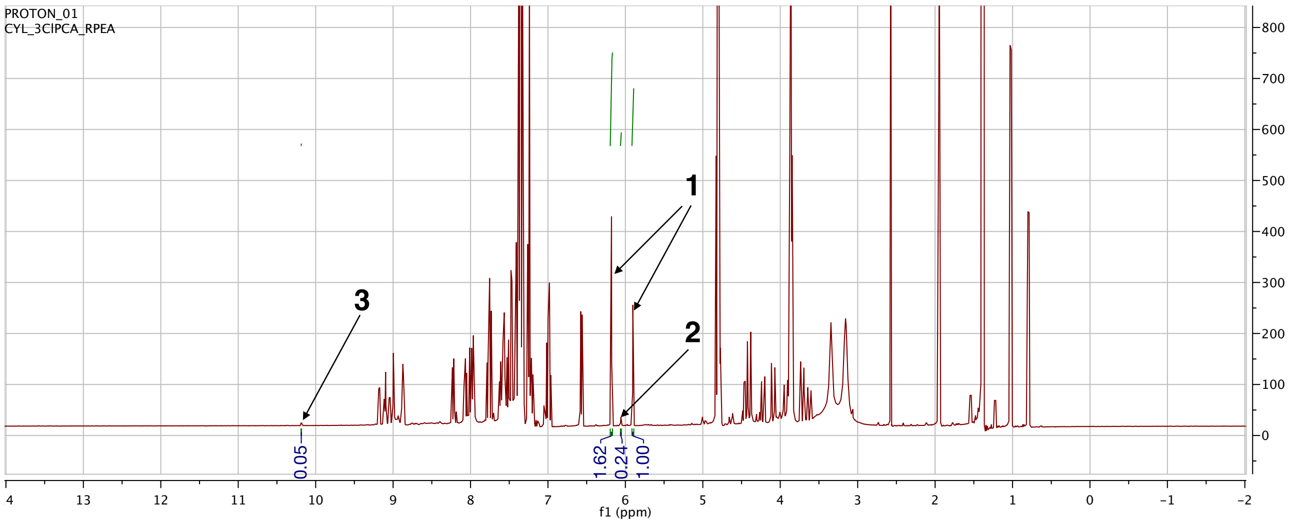

6-chloropyridine-2-carbaldehyde

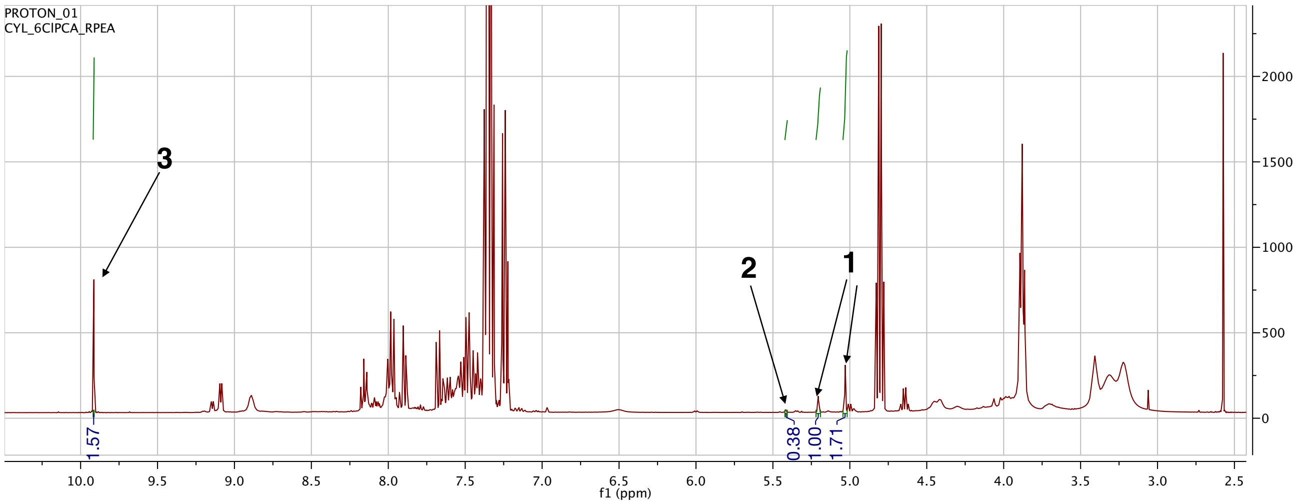

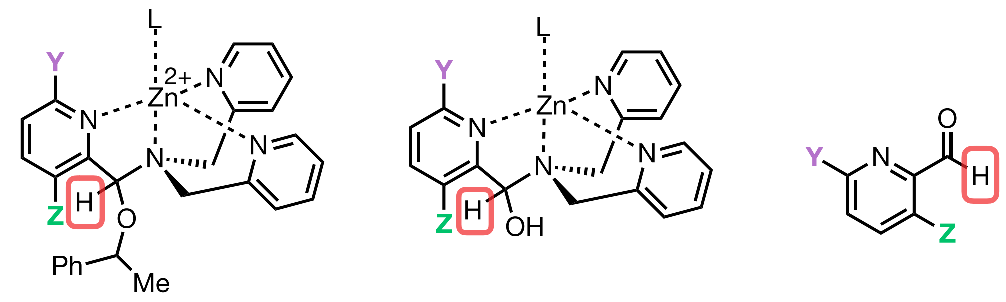

1 **2** **3**

3-bromopyridine-2-carbaldehyde

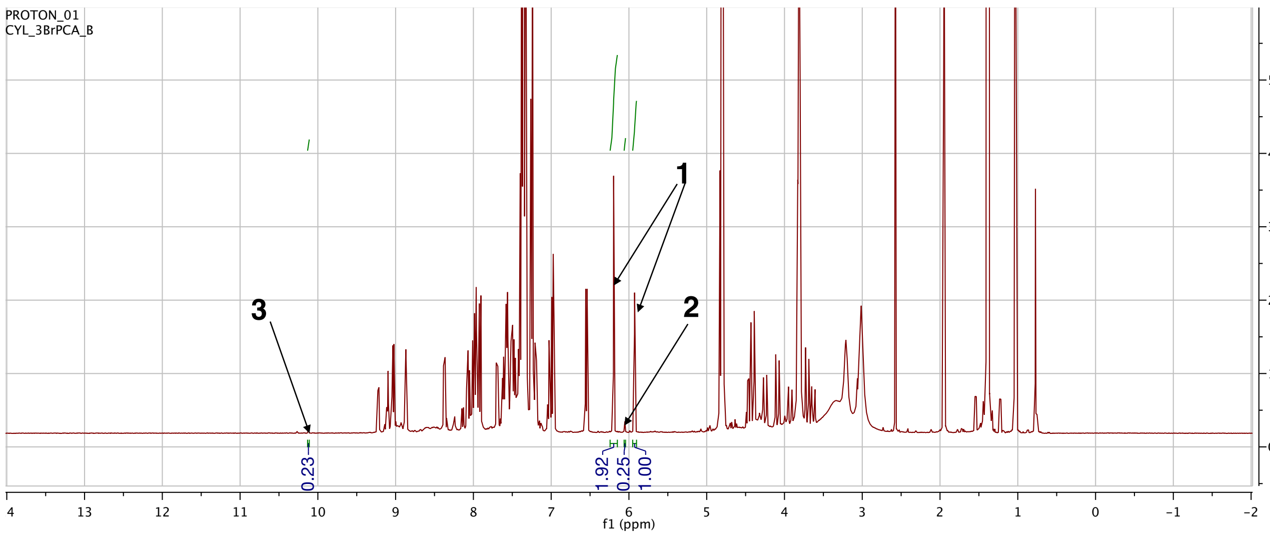

6-bromopyridine-2-carbaldehyde

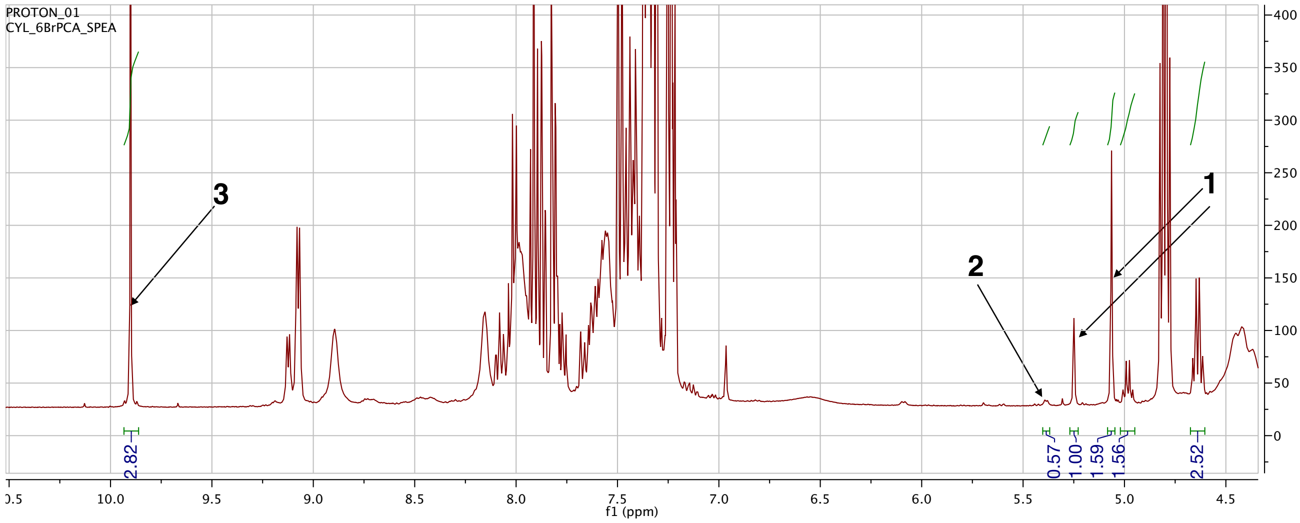

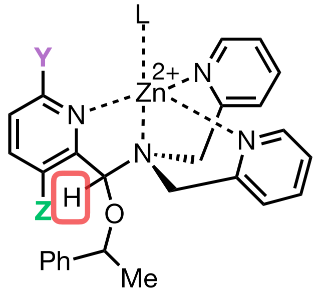

1

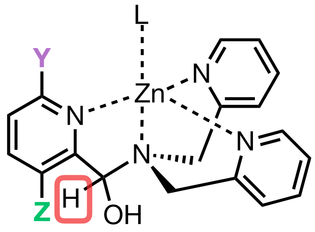

2

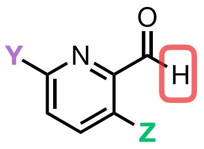

3

3-methoxypyridine-2-carbaldehyde

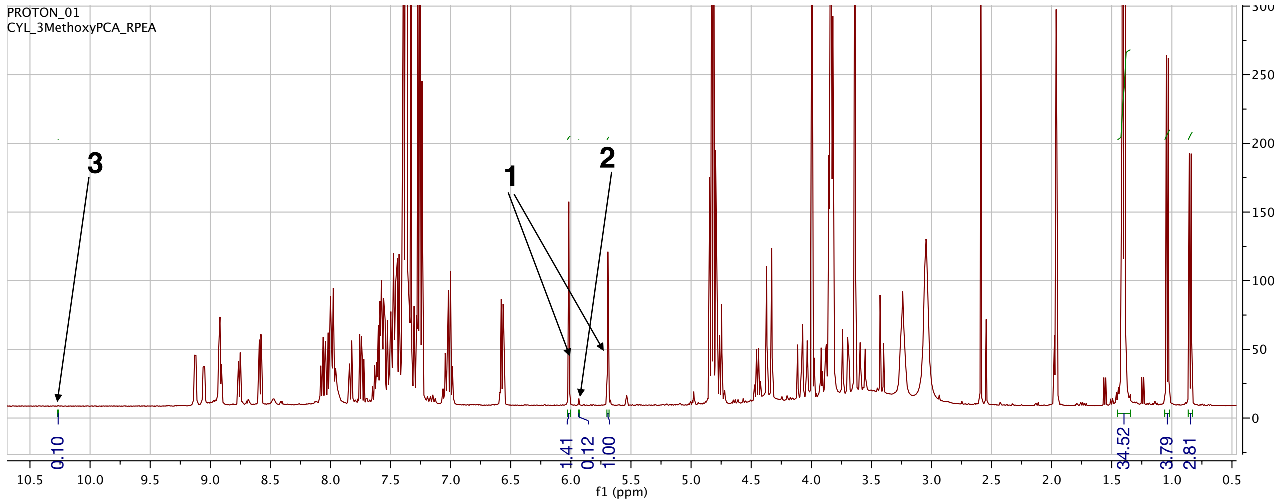

6-methoxypyridine-2-carbaldehyde

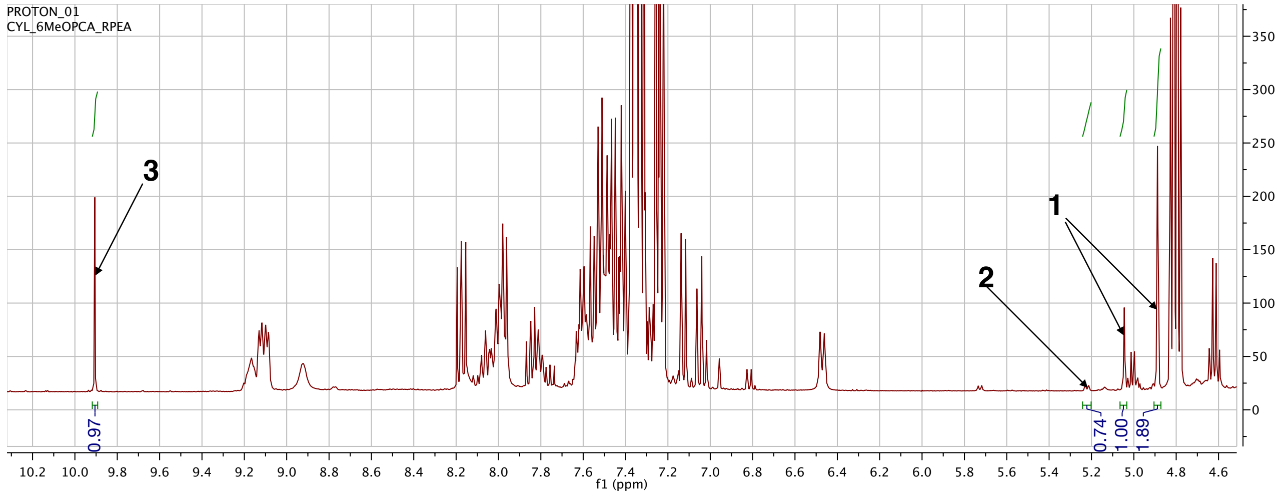

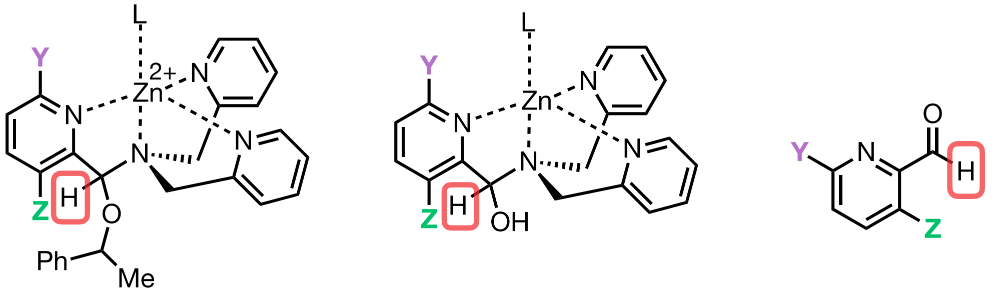

1 **2** **3**

3-methylpyridine-2-carbaldehyde

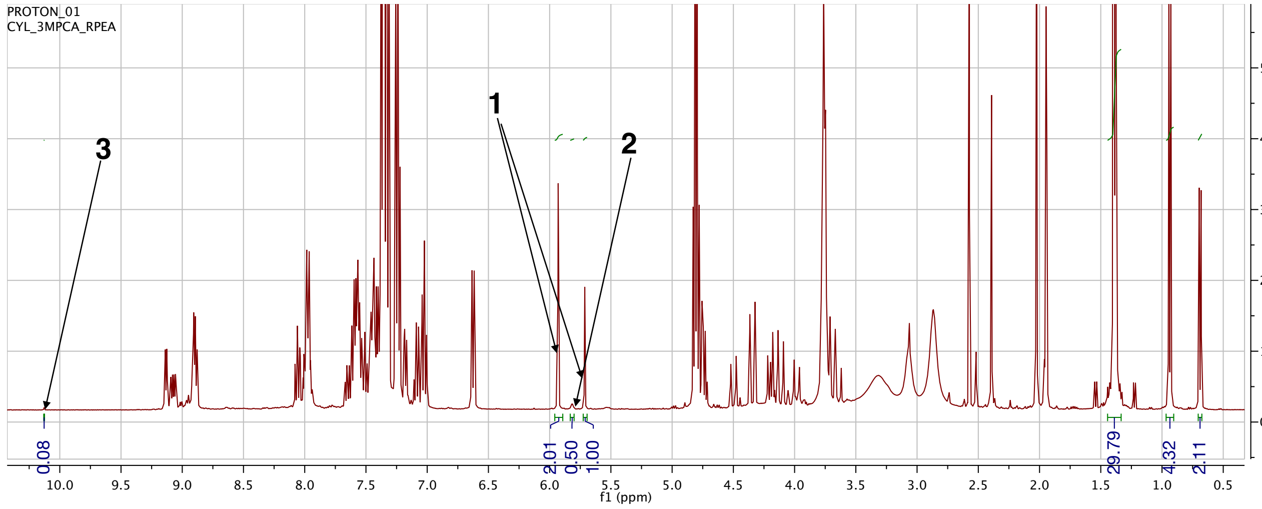

6-methylpyridine-2-carbaldehyde

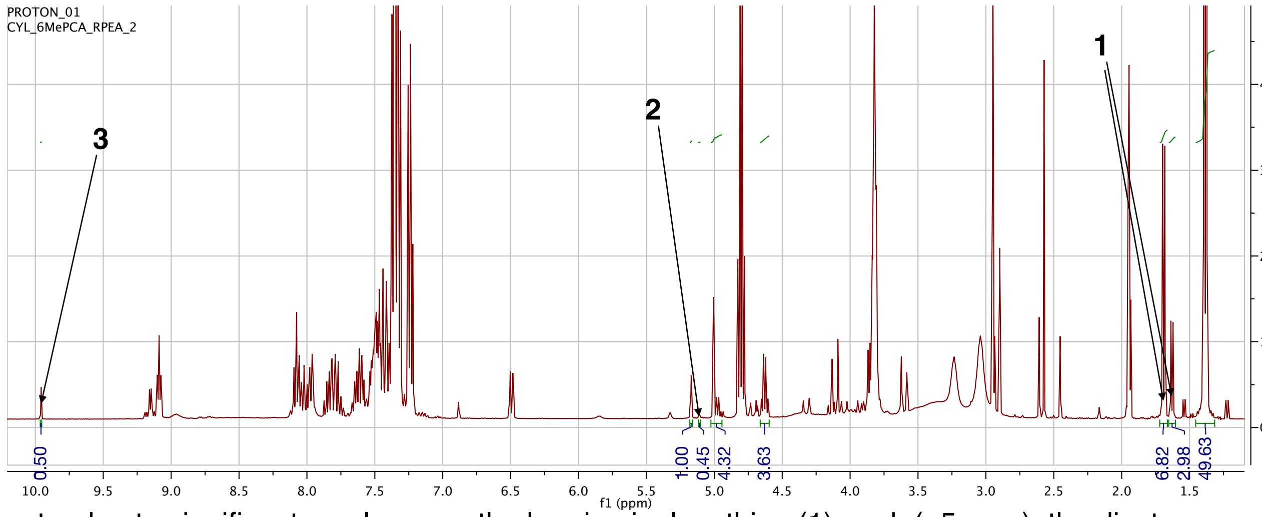

note: due to significant overlap near the hemiaminal methine (1) peak (~5 ppm), the diastereomeric ratio was determined using the diastereomeric methyl peaks (~1.7 ppm).

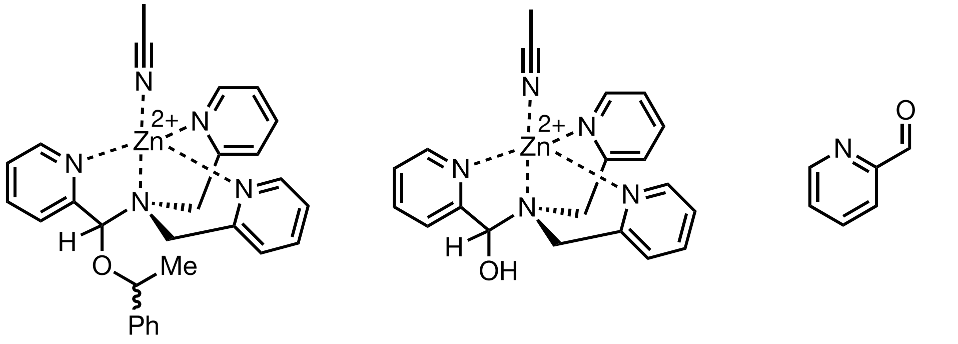

1 2 3

2-pyridinecarbaldehyde

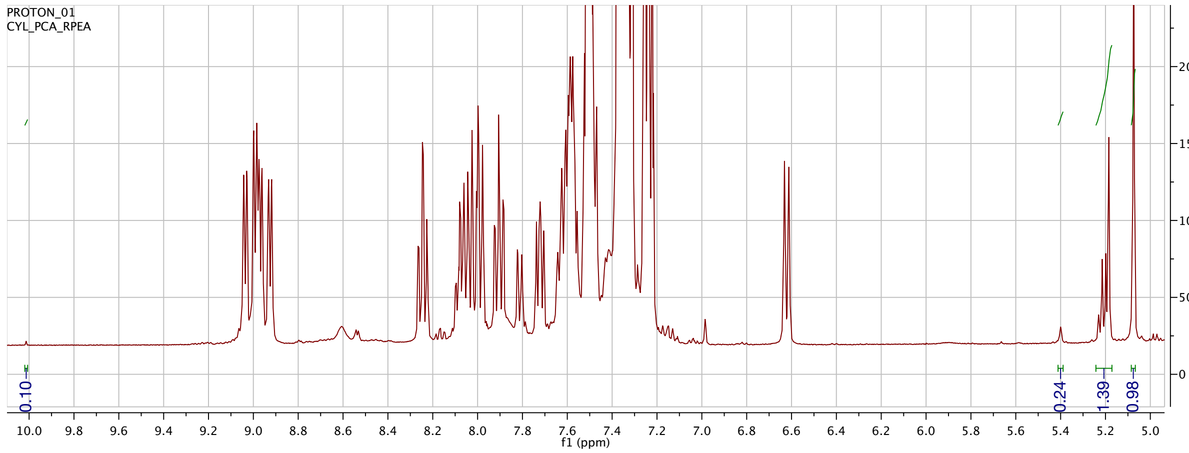

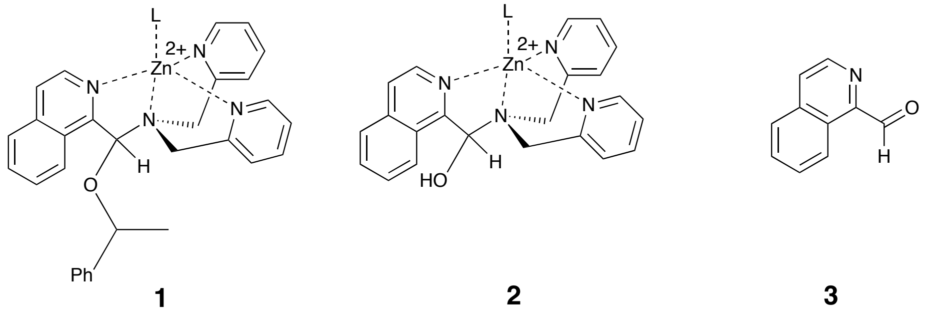

1 2 3

isoquinoline-1-carbaldehyde

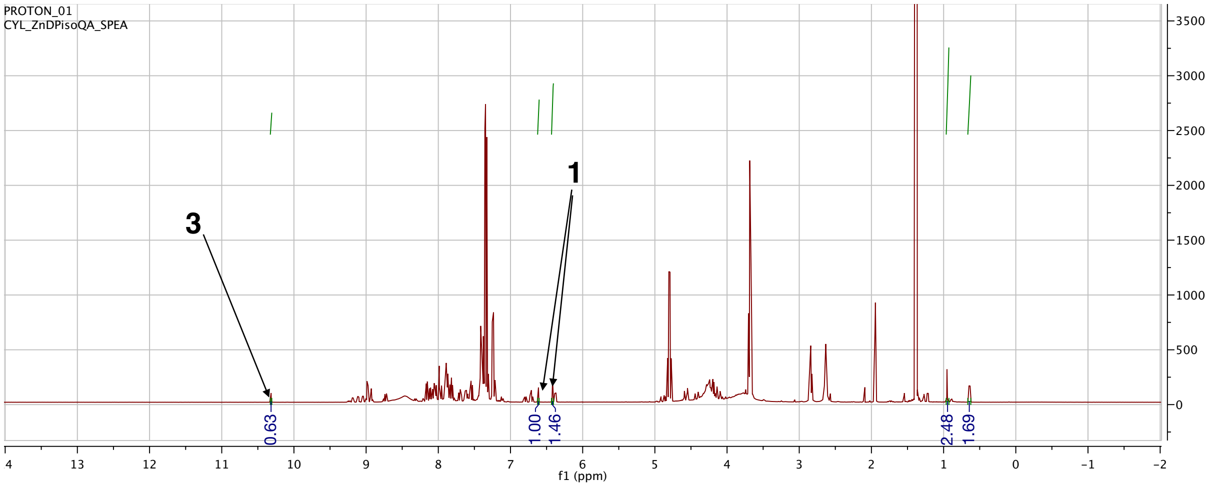

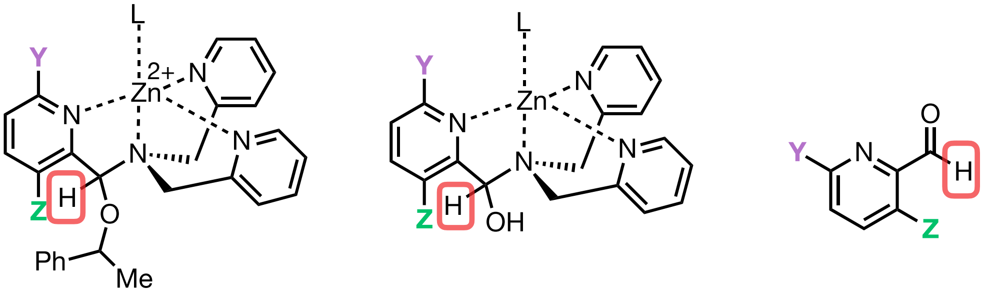

1 **2** **3**

3-fluoropyridine-2-carbaldehyde

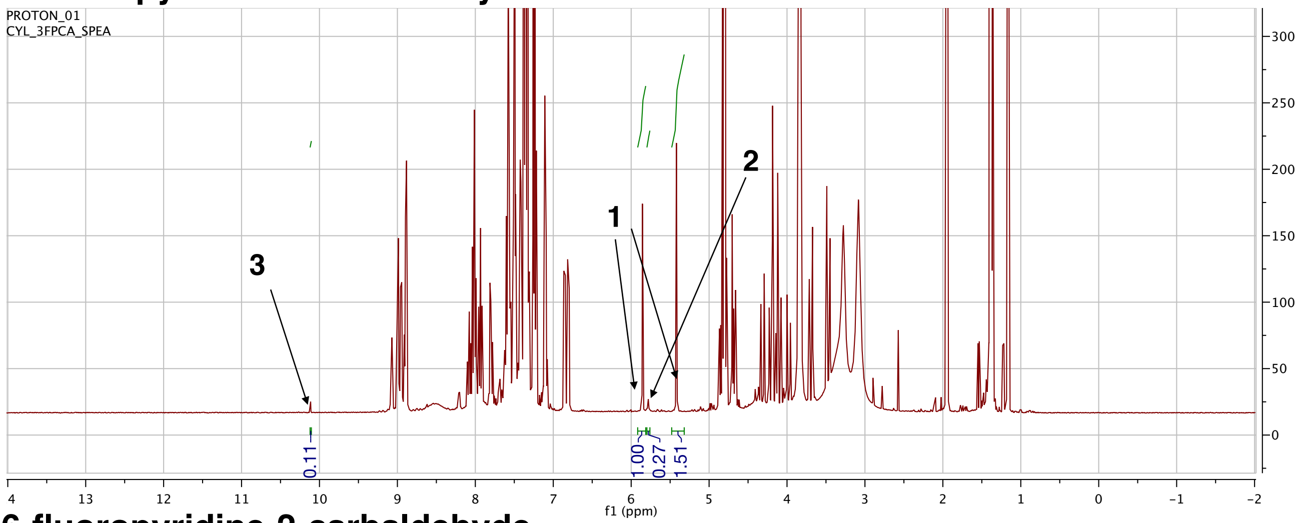

6-fluoropyridine-2-carbaldehyde

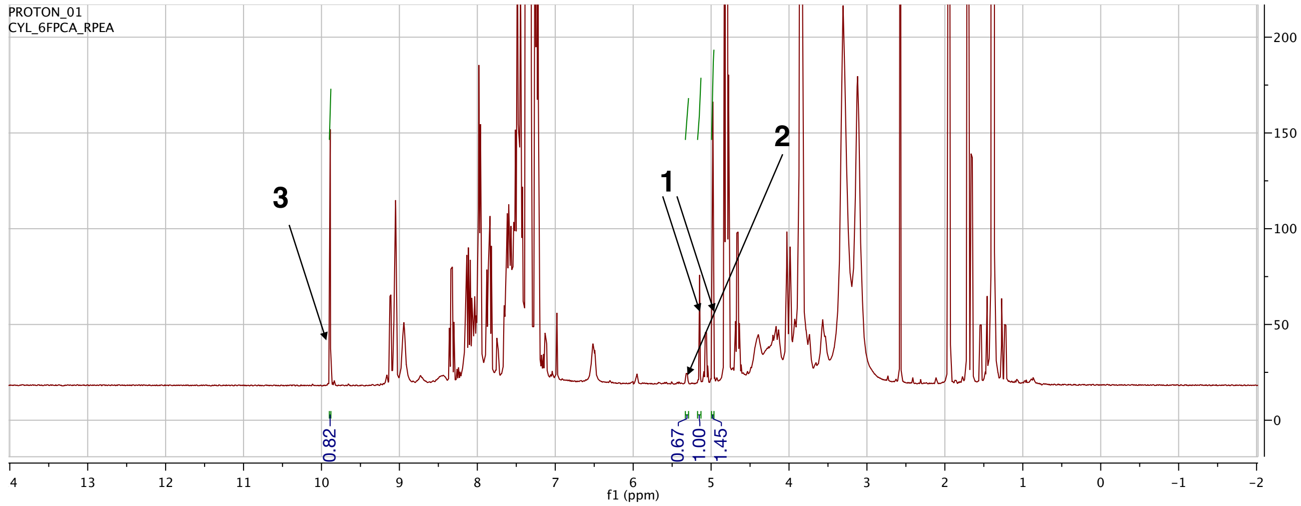

Calculating the Extend of Formation (Yield) of Hemiaminal Ether Assemblies

Yield is defined as the extend of hemiaminal ether formation with the pyridinecarbaldehyde as the limiting reagent. ^{1}H NMR was used for calculating the yield. The spectra were taken at 35 mM with no visible precipitation. The proton peaks of interest are identified, integrated, then used to calculate the yield. Using the following ^{1}H NMR spectrum as an example, the aldehyde (10.2 ppm), hemiaminal methine (6.1 ppm), and hemiaminal ether diastereomeric methines (5.9 ppm and 6.3 ppm) were identified and integrated. The integrated values are used to calculate the yield.

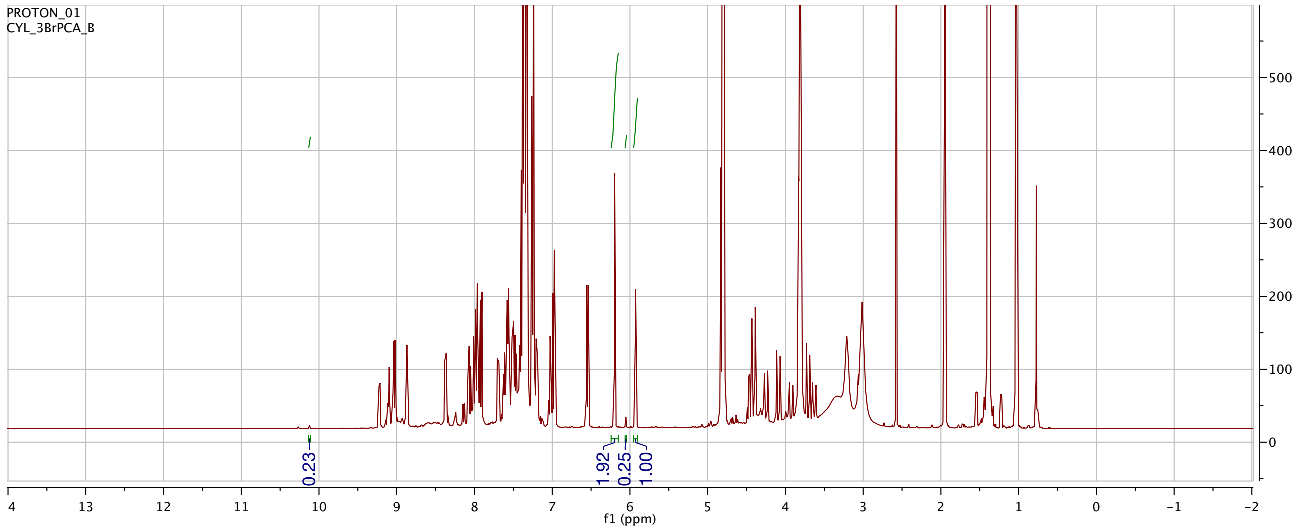

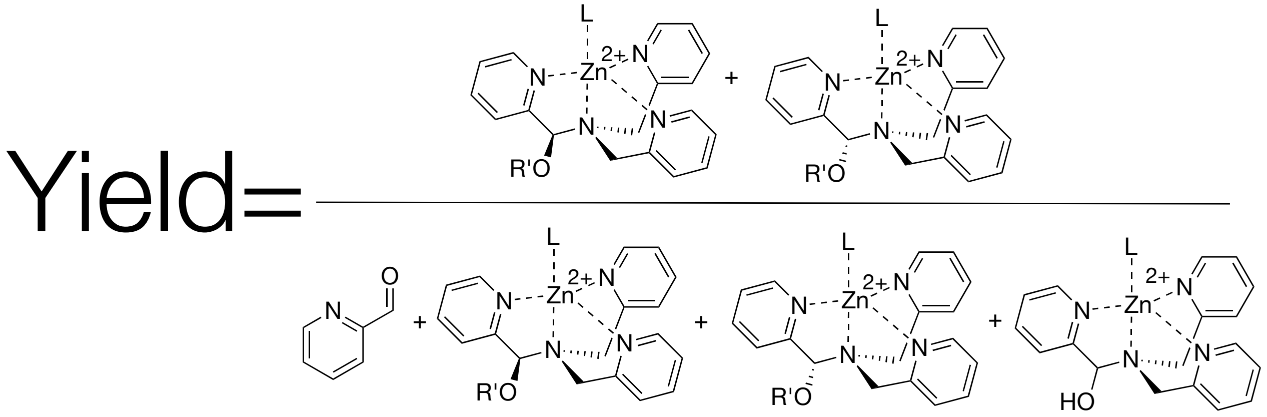

CD Studies of 2PA' Ligands

CD spectra were taken with Jasco J-815 CD Spectrometer with Starna Type 21 1-mm quartz cuvette at 25 °C in acetonitrile at 175 µM . The assemblies were formed with protocols described in the main text of the article. CD spectra of assemblies with varying 2PA' ligand are shown below.

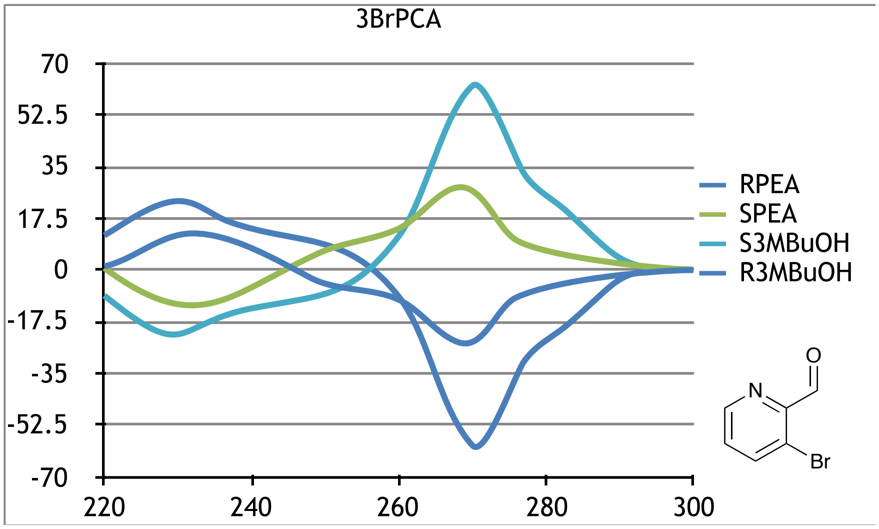

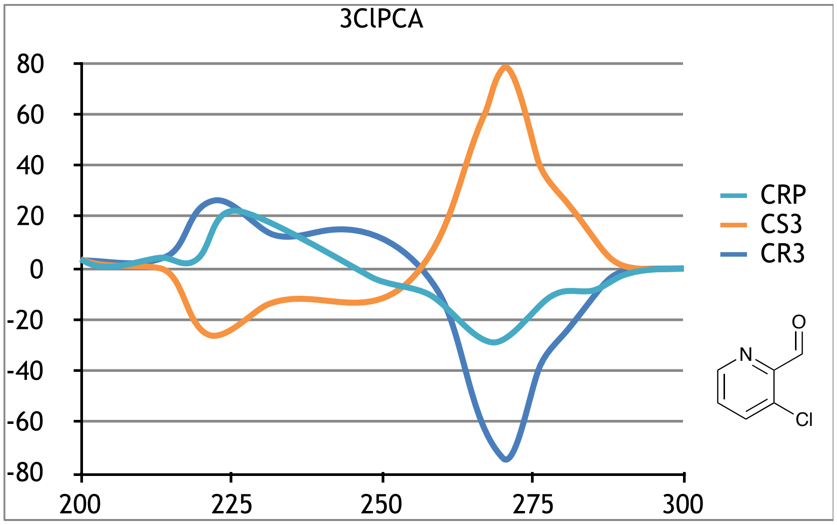

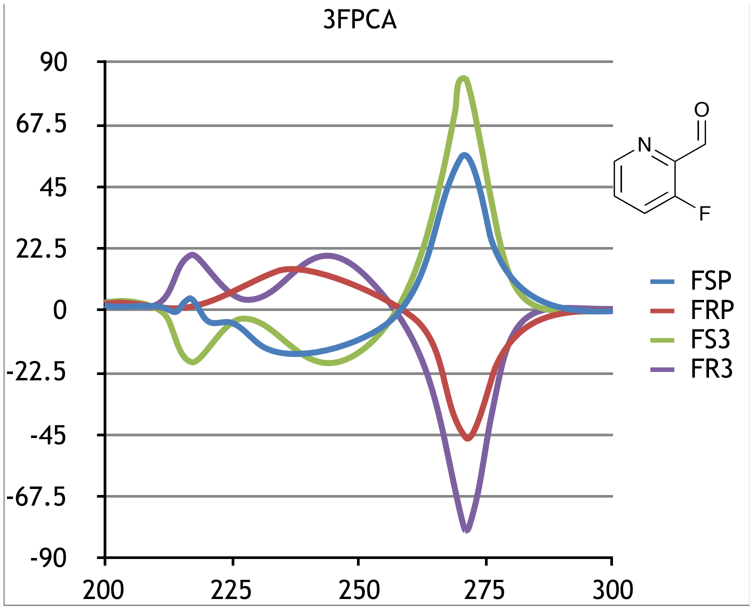
3FPCA
90
67.5
45
22.5
0
-22.5
-45
-67.5
-90
200
225
250
275
300
O
N
F
FSP
FRP
FS3
FR3

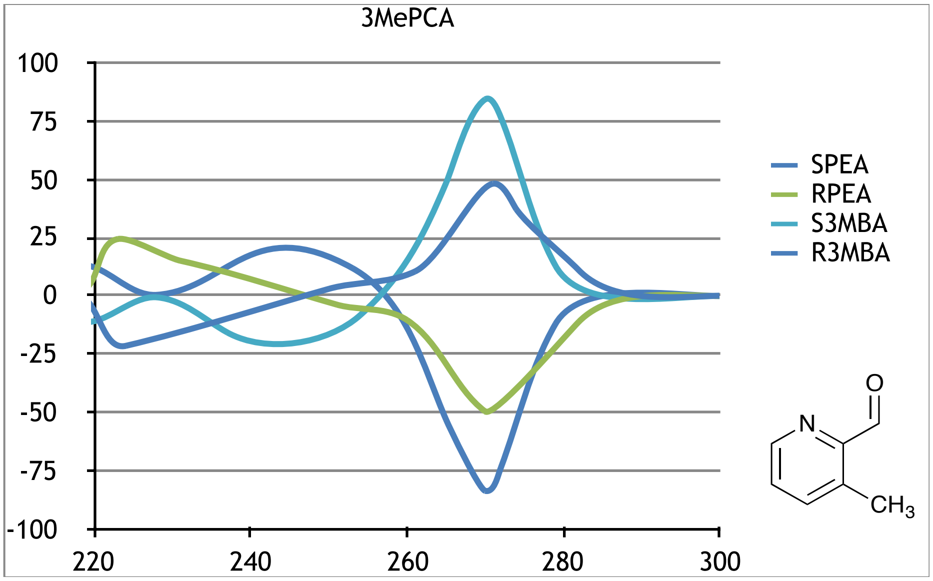
3MePCA
100
75
50
25
0
-25
-50
-75
-100
220
240
260
280
300
SPEA
RPEA
S3MBA
R3MBA
O
N
CH3

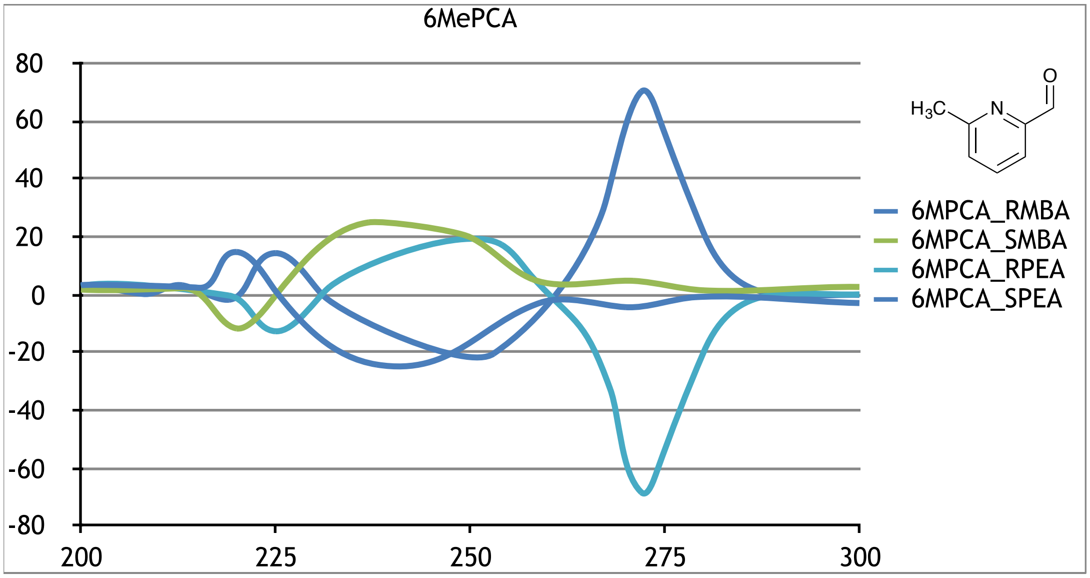
6MePCA
80
60
40
20
0
-20
-40
-60
-80
200
225
250
275
300
H3C
N
O
6MPCA_RMBA
6MPCA_SMBA
6MPCA_RPEA
6MPCA_SPEA

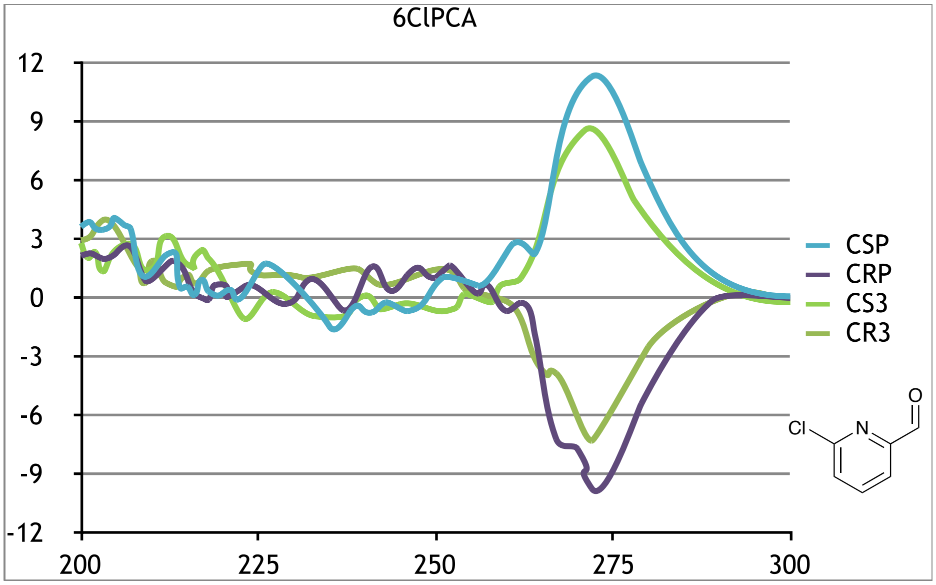
6ClPCA
12
9
6
3
0
-3
-6
-9
-12
200
225
250
275
300
CSP
CRP
CS3
CR3
Cl
N
O

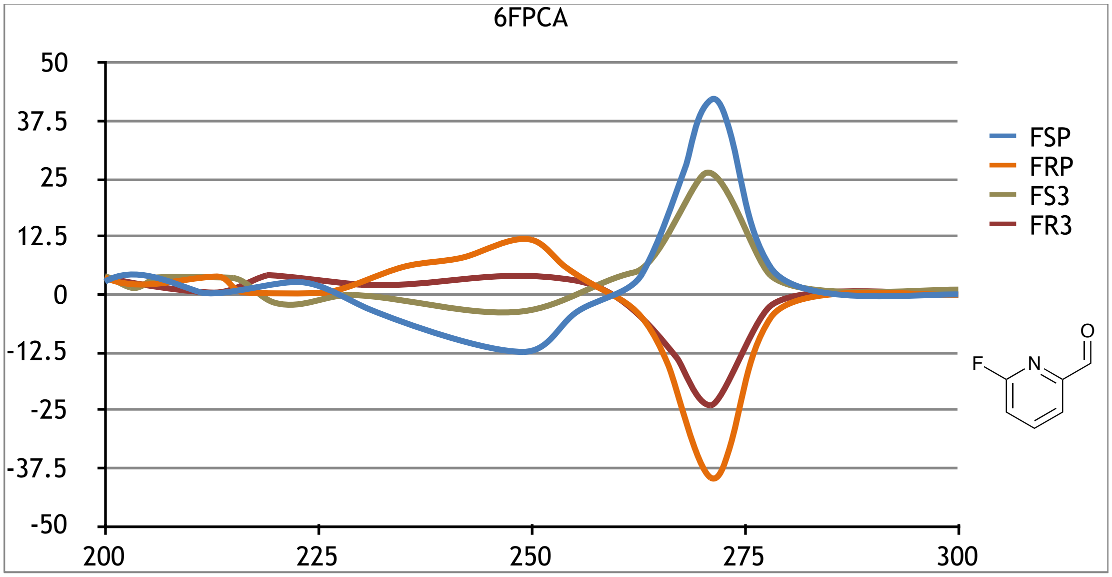
6FPCA
50
37.5
25
12.5
0
-12.5
-25
-37.5
-50
200
225
250
275
300
FSP
FRP
FS3
FR3
F
N
O

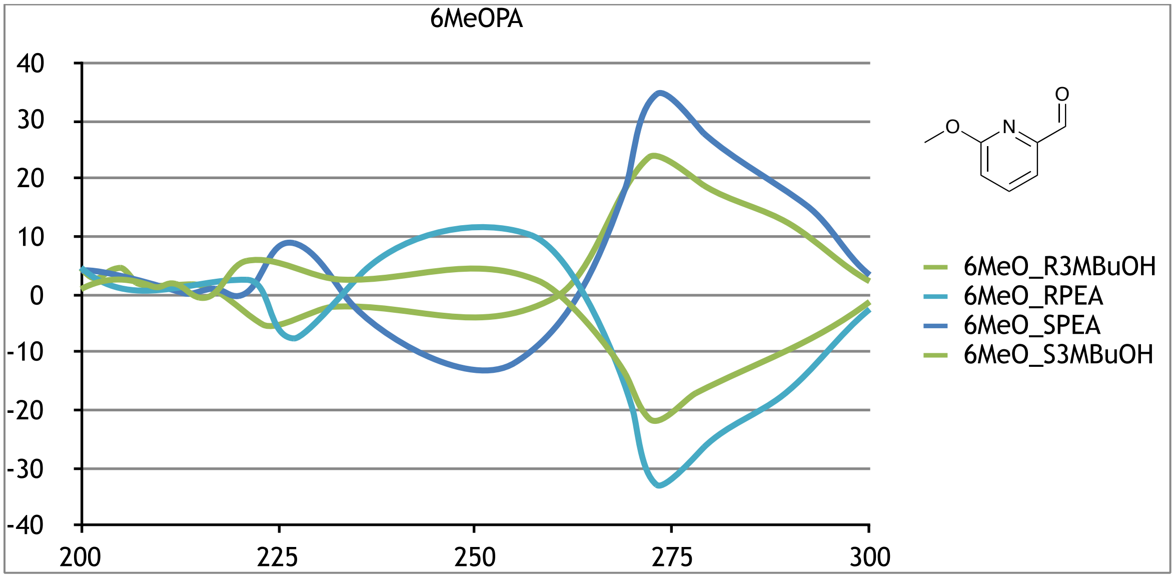
6MeOPA
40
30
20
10
0
-10
-20
-30
-40
200
225
250
275
300
O
N
O
6MeO_R3MBuOH
6MeO_RPEA
6MeO_SPEA
6MeO_S3MBuOH

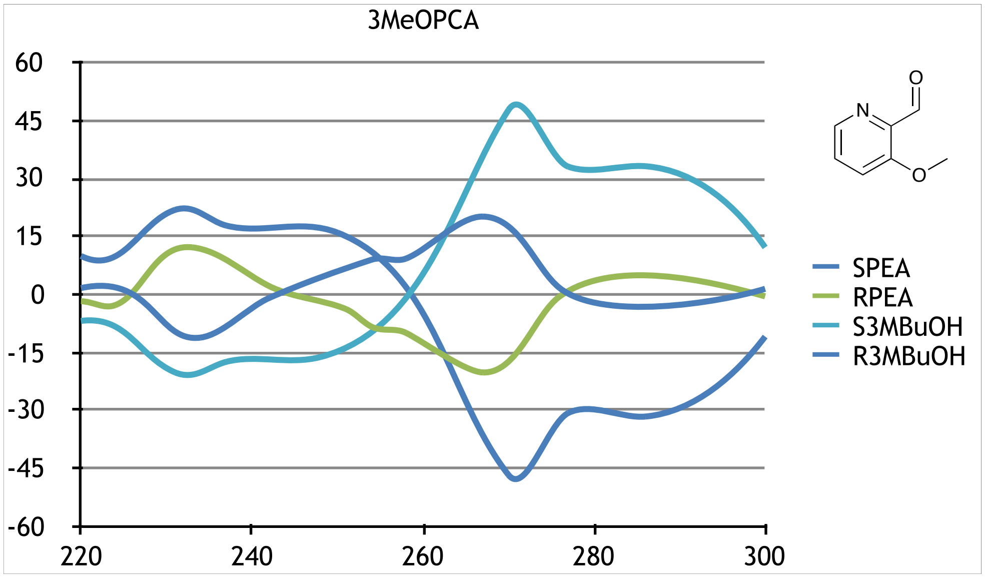
3MeOPCA
60
45
30
15
0
-15
-30
-45
-60
220
240
260
280
300
O
N
O
SPEA
RPEA
S3MBuOH
R3MBuOH

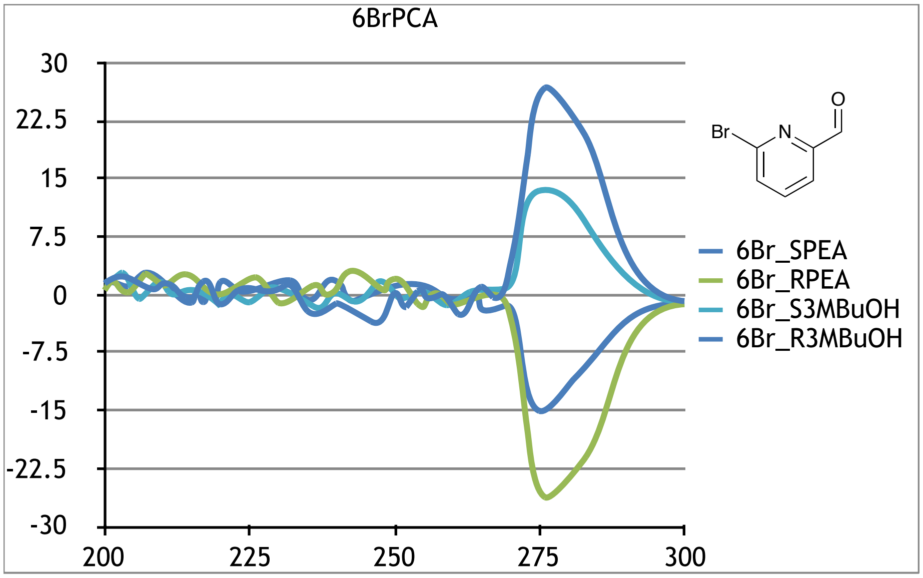
6BrPCA
30
22.5
15
7.5
0
-7.5
-15
-22.5
-30
200
225
250
275
300
Br
N
O
6Br_SPEA
6Br_RPEA
6Br_S3MBuOH
6Br_R3MBuOH

3-Methylpyridine-2-carbaldehyde Assembly Alcohol ^{1}H NMR Results

The assembly was formed with described procedure in the main text in acetonitrile at 50 mM, and the *dr* was derived from ^{1}H NMR spectra. Only the diastereomeric methine peaks are integrated in the following spectra.

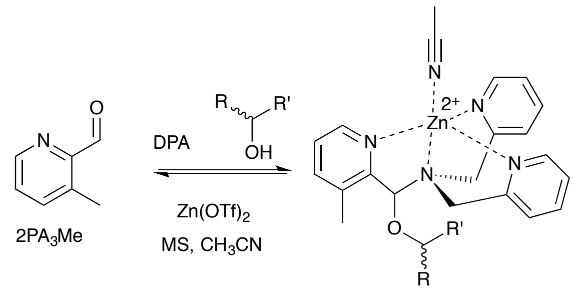

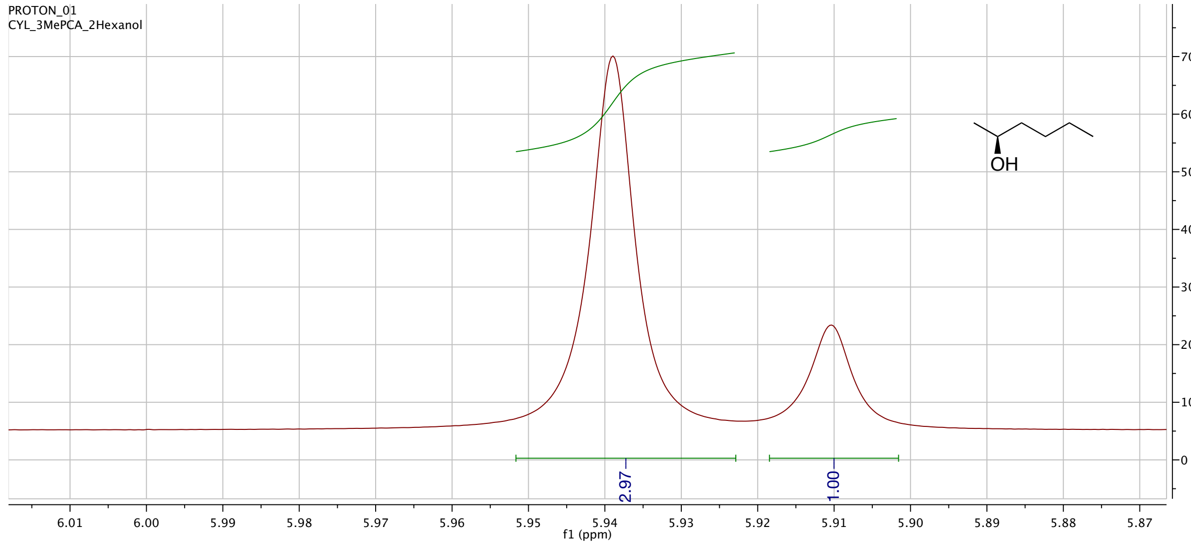

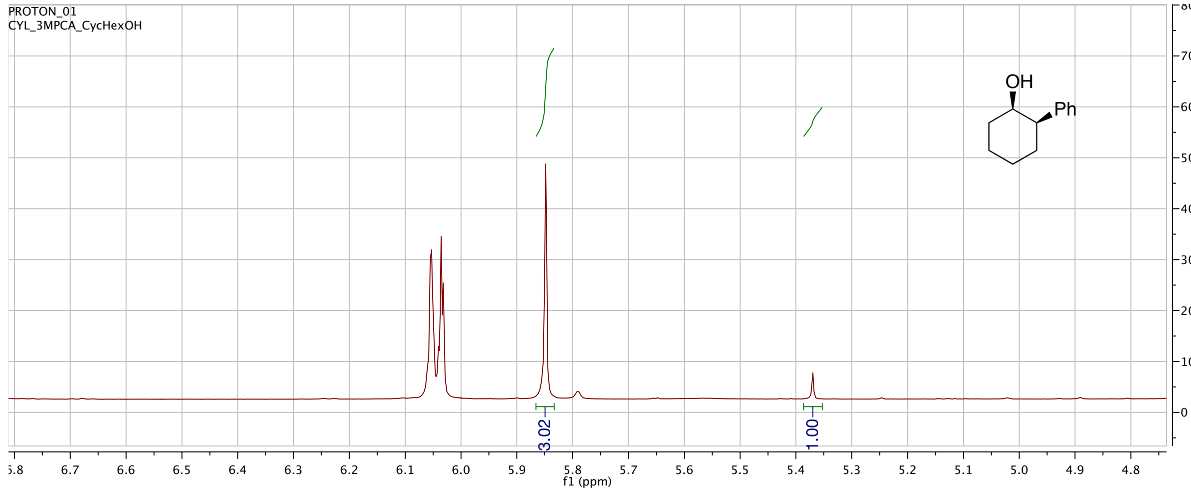

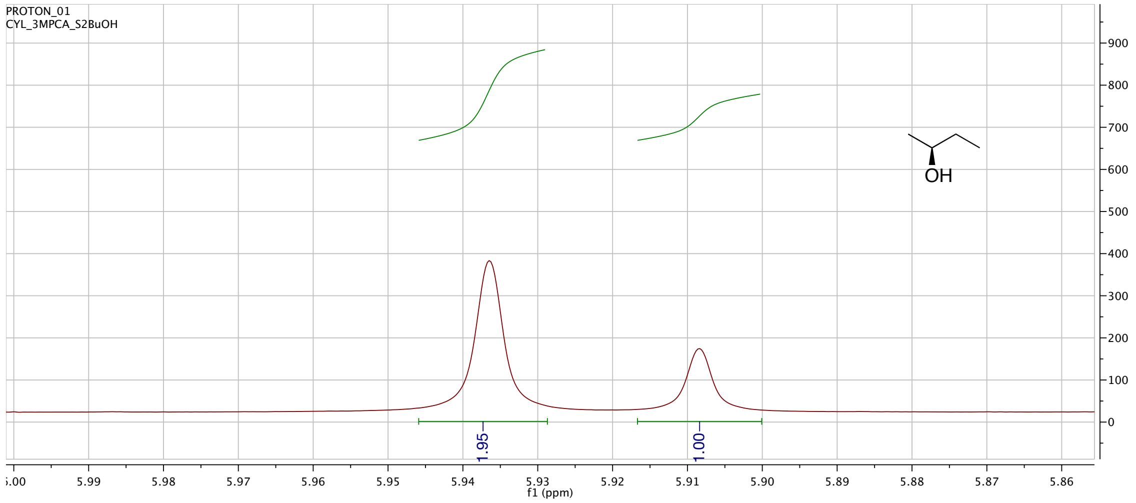
PROTON_01
CYL_3MPCA_S2BuOH
OH
1.95
1.00
f1 (ppm)

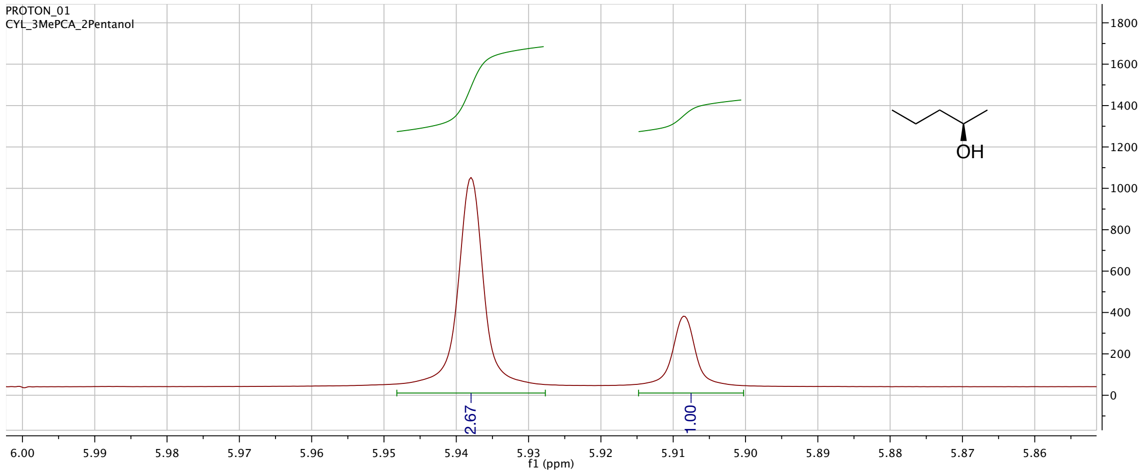
PROTON_01
CYL_3MePCA_2Pentanol
OH
2.67
1.00
f1 (ppm)

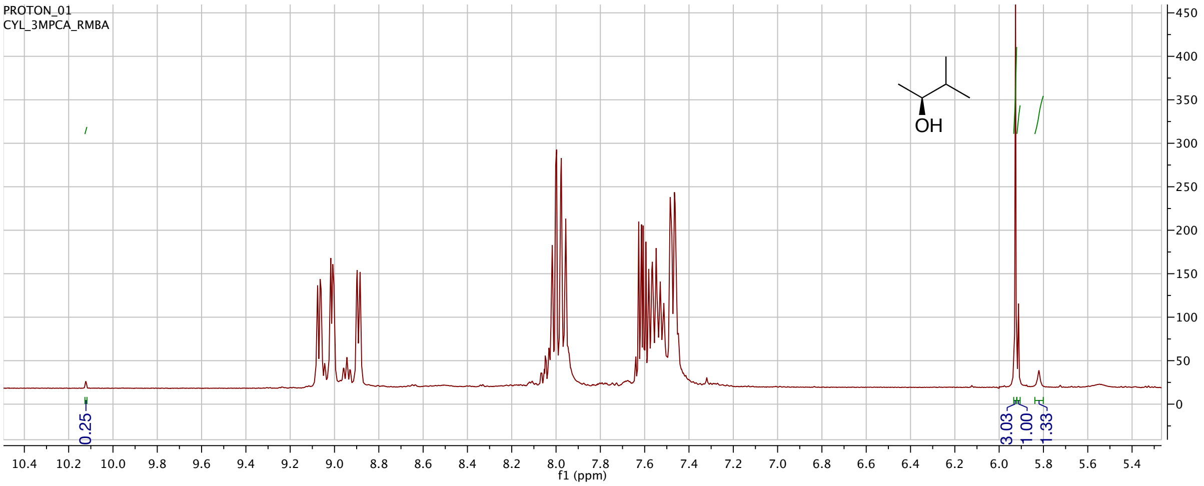
PROTON_01
CYL_3MPCA_RMBA
OH
0.25
3.03
1.00
1.33
f1 (ppm)

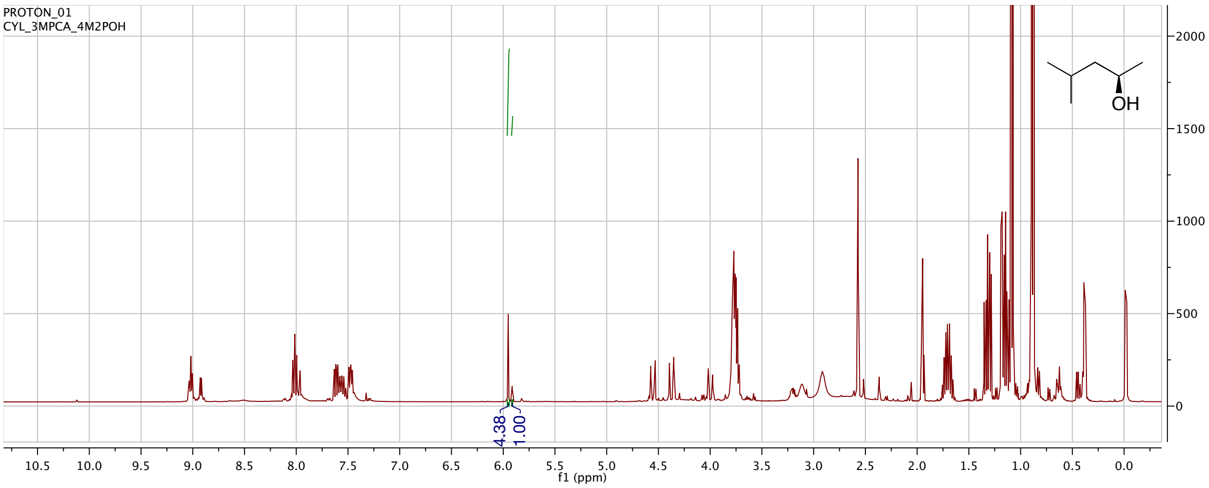
PROTON_01
CYL_3MPCA_4M2POH
OH
4.38
1.00
f1 (ppm)

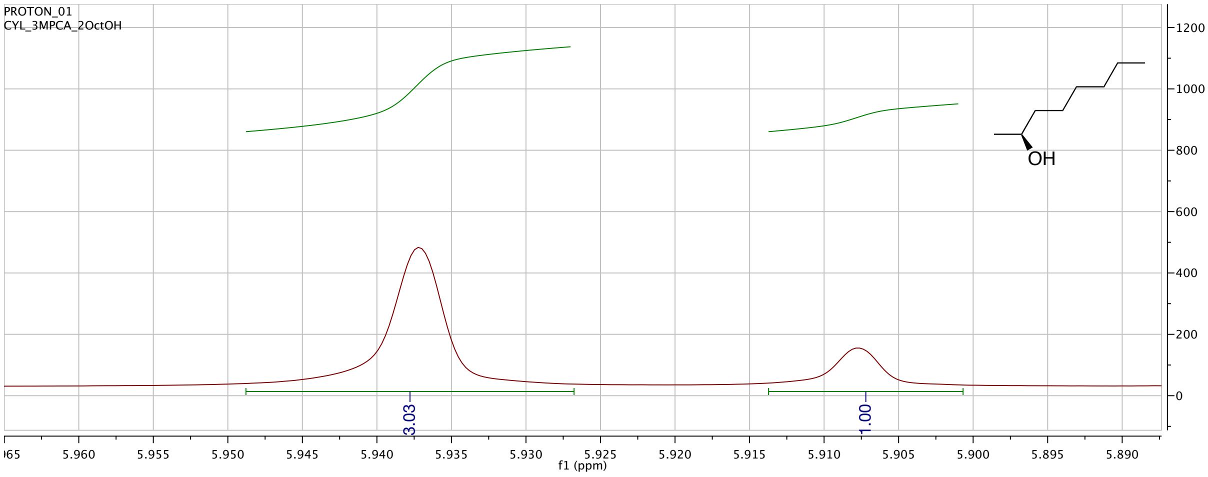
PROTON_01
CYL_3MPCA_2OctOH
OH
3.03
1.00
f1 (ppm)

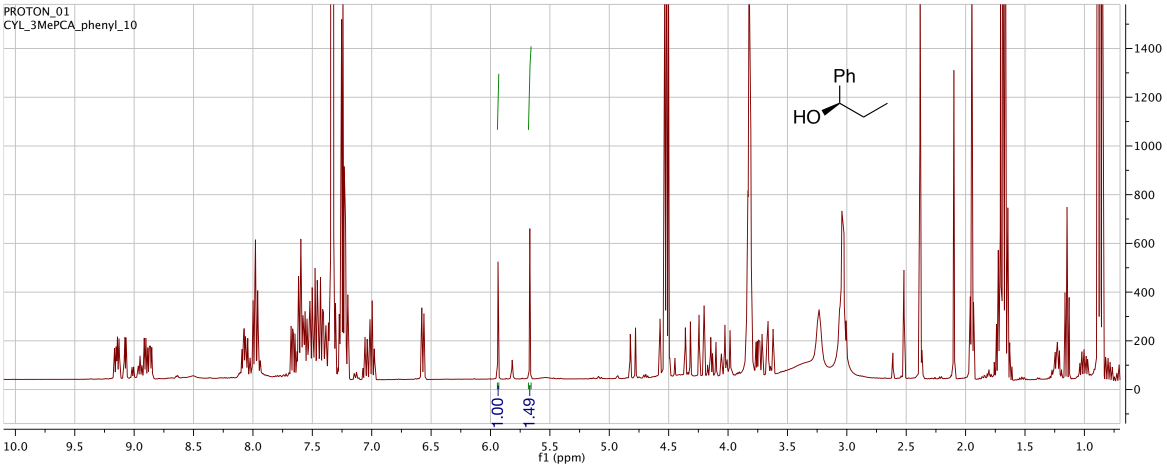
PROTON_01
CYL_3MePCA_phenyl_10
Ph
HO
1.00
1.49
f1 (ppm)

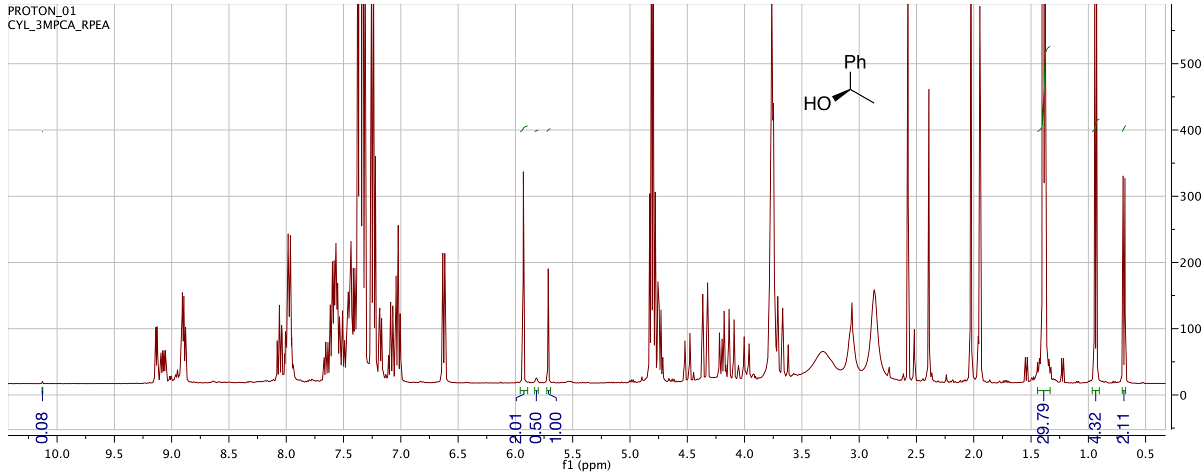

PROTON_01
CYL_3MPCA_RPEA
Ph
HO
0.08
2.01
0.50
1.00
29.79
4.32
2.11
f1 (ppm)

Synthesis of isoquinoline-1-carboxyaldehyde

The synthesis followed literature procedure by Long *et al.*[42] 1-Methylquinolin (0.300 g, 2.1 mmol) and SeO_2 (0.323 g, 2.91 mmol) were dissolved in 1,4-dioxane (20 mL) and refluxed for 1.5 hour under nitrogen. The reaction mixture was filtered through Celite and concentrated *in vacuo*. Product was purified using column chromatography (silica; hexanes: EtOAc, 5:1 v/v) to give 72% yield.

^{1}H-NMR (400MHz, CD_3CN): δ (ppm) 7.76 (m, 2H), 7.90 (m, 2H), 8.73 (d, 1H), 9.27 (m, 1H), 10.35 (s, 1H).

Synthesis of phenanthridine-6-carbaldehyde

This synthesis procedure followed the protocol by Pierre and Cladwell.[38,40] 2-Aminobiphenyl (1.01 g, 5.97 mmol) was dissolved in acetic anhydride (1.52 g, 14.5 mmol) and stirred for 10 minutes. One equivalent of acetic anhydride (0.59 g, 5.97 mmol) was added to the reaction followed by 30 minutes stirring. The reaction was then poured over ice where precipitation was observed. The solids were collected by vacuum filtration and washed with water. The solids were left dried in air overnight yielding 1.12 g (89% yield). The solid (0.701 g, 3.32 mmol) was then dissolved in polyphosphoric acid (10 mL) and stirred at 150°C for 2.5 hours. The reaction mixture was then cooled to 0°C and basified using NaOH (1M) to pH 10. The reaction was extracted with DCM (4*30 mL), dried ($MgSO_4$), then concentrated in vacuo. The product was purified with column chromatography (silica; DCM:MeOH, MeOH starting at 0% and end at 10%) to give a white/orange solid. The solid was then stirred with EtOAc (20mL) with SeO_2 (0.516 g, 4.65 mmol) followed by 12 hours refluxing. The reaction mixture was extracted with water (pH 4, 50mL*4) followed by basifying the aqueous layer to pH 10. The basified extract was extracted with chloroform (50 mL*4). The organic layer was dried ($MgSO_4$) and concentrated to give a white solid (0.362 g, 53% yield).

^{1}H-NMR (400MHz, CD_3CN): δ (ppm) 7.78 (dd, 1H), 7.82 (m, 2H), 7.91 (dd, 1H), 8.32 (m, 1H), 8.63 (m, 1H), 8.68 (m, 1H), 9.43 (dd, 1H), 10.42 (s, 1H).

Synthesis of (2-pyridylmethyl) (2-quinolylmethyl)amine

This synthesis procedure followed the protocol published by Rompel and coworkers.[41] 2-quinolinecarboxyaldehyde (1.000g, 6.35 mmol) and 2-aminomethylpyridine (0.688g, 6.35 mmol) was mixed in MeOH (20 mL) with MS. The reaction was left stirring for 3 hours. Then, the reaction was filtered and cooled to 0°C followed by $NaBH_4$ (0.336 g, 8.89 mmol) addition. The reaction was then left stirring in room temperature overnight. The reaction was then acidified to pH 1 (2M HCl) and extracted with chloroform (30 mL*4). The aqueous layer was basified to pH 10 (1M NaOH) and extracted with chloroform (30 mL*4). The organic layer was concentrated *in vacuo* to give a dark maroon oil. The crude product was then purified using column chromatography (alumina, 1% MeOH in DCM) to give 0.569 g of red oil (36% yield).

^{1}H-NMR (400MHz, CD_3CN): δ (ppm) 3.94 (s, 2H), 4.07 (s, 2H), 7.02 (dd, 1H), 7.24 (d, 1H), 7.32 (td, 1H), 7.48 (td, 1H), 7.56 (td, 1H), 7.65 (dd, 1H), 7.96 (t, 1H), 8.46 (dd, 1H).

Synthesis of (2-isoquinolylmethyl) (2-pyridylmethyl)amine

This synthesis procedure followed closely to the protocol published by Rompel and coworkers.[41] 2-isoquinolinecarboxyaldehyde (0.486 g, 3.05 mmol) and 2-aminomethylpyridine (0.330 g, 3.05 mmol) was mixed in MeOH (20 mL) with MS. The reaction was left stirring for 3 hours. Then, the reaction was filtered and cooled to 0°C followed by $NaBH_4$ (0.115 g, 3.05 mmol) addition. The reaction was then left stirring in room temperature overnight. The reaction was then acidified to pH 1 (2M HCl) and extracted with chloroform (30 mL*4). The aqueous layer was basified to pH 10 (1M NaOH) and extracted with chloroform (30 mL*4). The organic layer was concentrated *in vacuo* to give a dark maroon oil (not purified).

Synthesis of bis(2-quinolylmethyl)amine

2-chloromethylquinoline (5.026 g, 23.47 mmol) was dissolved in DI water and then treated with 1 equivalent of NaOH (1 M). The precipitate was collected and recrystallized in hexanes. The recrystallized product was then dissolved in DMF (30mL), mixed with NaN_3 (7.631g, 117.4 mmol), and left stirring overnight. The reaction mixture was washed with water (30 mL) and extracted with EtOAc (30 mL *3). The organic extract was then concentrated to give about 20 mL of the product with EtOAc. Pd/C (10 wt%, 0.249 g, 2.34 mmol) was then added to the mixture, and the reaction was left stirring overnight under H_2 in atmosphere. The product mixture was concentrated *in vacuo* to give a purple oil. This purple oil was then mixed with 2-quinolinecarboxyaldehyde (3.689g, 23.47 mmol) and MS in MeOH (20 mL) for 3 hours. The reaction was then filtered, cooled to 0 °C, followed by $NaBH_4$ (1.154g, 30.51 mmol) addition, and left stirring at room temperature overnight. The reaction was acidified to pH 1 (2M HCl) and extracted with chloroform (30 mL*4). The aqueous layer was then basified to pH 10 (1M NaOH) and extracted with chloroform (30 mL*4). The organic layer was dried ($MgSO_4$), filtered, and concentrated *in vacuo.* The crude product was purified using column chromatography (alumina, 1% MeOH in DCM) to yield 1.474 g (21% yield).

^{1}H-NMR (400MHz, CD_3CN): δ (ppm) 4.91 (s, 4H), 7.28 (d, 2H), 7.53 (dd, 2H), 7.72 (dd, 2H), 7.82 (dd, 2H), 8.07 (d, 2H), 8.14 (d, 2H).

Glossary

**2PA**	pyridine-2-carbaldehyde
**DPA**	di-(2-picolyl)amine
**2PA'**	3 or 6 substituted pyridine-2-carbaldehyde derivatives
**QA**	quinoline-2-carbaldehyde
**PNA**	phenanthridine-6-carbaldehyde
**IQA**	isoquinoline-1-carbaldehyde
**$2PA^{3}$**	3 substituted pyridine-2-carbaldehyde
**$2PA^{6}$**	6 substituted pyridine-2-carbaldehyde
**L**	axial ligand to the Zn multicomponent assembly
**$2PA'^{F}$**	3 or 6 fluoro substituted pyridine-2-carbaldehyde
**$2PA'^{Cl}$**	3 or 6 chloro substituted pyridine-2-carbaldehyde
**$2PA'^{Br}$**	3 or 6 bromo substituted pyridine-2-carbaldehyde
**$2PA'^{MeO}$**	3 or 6 methoxy substituted pyridine-2-carbaldehyde
**$2PA'^{Me}$**	3 or 6 methyl substituted pyridine-2-carbaldehyde
**DPHA**	methyl-6-hydroxy-2,6-diphenylhexanoate
